# Development of a High-Affinity Antibody against the Tumor-Specific and Hyperactive 611-p95HER2 Isoform

**DOI:** 10.3390/cancers14194859

**Published:** 2022-10-05

**Authors:** Esmaeil Dorraji, Elin Borgen, Dario Segura-Peña, Puneet Rawat, Eva Smorodina, Claire Dunn, Victor Greiff, Nikolina Sekulić, Hege Russnes, Jon Amund Kyte

**Affiliations:** 1 Department of Cancer Immunology, Institute for Cancer Research, Oslo University Hospital, 0379 Oslo, Norway; 2 Department of Pathology, Oslo University Hospital, 0379 Oslo, Norway; 3 Centre for Molecular Medicine Norway (NCMM), Nordic EMBL Partnership, Faculty of Medicine, University of Oslo, 0318 Oslo, Norway; 4 Department of Immunology, University of Oslo and Oslo University Hospital, 0372 Oslo, Norway; 5 Department of Chemistry, University of Oslo, 0371 Oslo, Norway; 6 Department of Cancer Genetics, Institute for Cancer Research, Oslo University Hospital, 0379 Oslo, Norway; 7 Department of Clinical Cancer Research, Oslo University Hospital, 0379 Oslo, Norway

**Keywords:** HER2, p95HER2, antibody, tumor-specific, breast cancer, cancer stem cells, HDX-MS, epitope and paratope mapping, docking

## Abstract

**Simple Summary:**

In the present study, we addressed the unmet need for a molecular antibody (mAb) with high affinity and specificity against a truncated hyperactive isoform of human epidermal growth factor receptor 2 (HER2), called 611-carboxy terminal fragment (CTF)-p95HER2. Patients with p95HER2+ breast cancer are at risk of developing metastatic breast cancer with a poor prognosis and resistance to therapies targeting full-length HER2. We have generated a mAb named Oslo-2, which react specifically with 611-CTF-p95HER2 and has a high affinity. We also characterized the antigenic determinant (epitope) on the p95HER2 protein and the antigen-binding site (paratope) on the Oslo-2 mAb. The antibody can be used to develop antibody- or cell-based therapies targeting p95HER2, as well as a diagnostic assay to identify p95HER2+ disease.

**Abstract:**

The expression of human epidermal growth factor receptor 2 (HER2) is a key classification factor in breast cancer. Many breast cancers express isoforms of HER2 with truncated carboxy-terminal fragments (CTF), collectively known as p95HER2. A common p95HER2 isoform, 611-CTF, is a biomarker for aggressive disease and confers resistance to therapy. Contrary to full-length HER2, 611-p95HER2 has negligible normal tissue expression. There is currently no approved diagnostic assay to identify this subgroup and no therapy targeting this mechanism of tumor escape. The purpose of this study was to develop a monoclonal antibody (mAb) against 611-CTF-p95HER2. Hybridomas were generated from rats immunized with cells expressing 611-CTF. A hybridoma producing a highly specific Ab was identified and cloned further as a mAb. This mAb, called Oslo-2, gave strong staining for 611-CTF and no binding to full-length HER2, as assessed in cell lines and tissues by flow cytometry, immunohistochemistry and immunofluorescence. No cross-reactivity against HER2 negative controls was detected. Surface plasmon resonance analysis demonstrated a high binding affinity (equilibrium dissociation constant 2 nM). The target epitope was identified at the N-terminal end, using experimental alanine scanning. Further, the mAb paratope was identified and characterized with hydrogen-deuterium-exchange, and a molecular model for the (Oslo-2 mAb:611-CTF-p95HER2) complex was generated by an experimental-information-driven docking approach. We conclude that the Oslo-2 mAb has a high affinity and is highly specific for 611-CTF-p95HER2. The Ab may be used to develop potent and safe therapies, overcoming p95HER2-mediated tumor escape, as well as for developing diagnostic assays.

## 1. Introduction

Breast cancer is the most prevalent malignancy in women [1] and is highly heterogeneous [2,3]. Around 20% of all breast cancer cases are classified as human epidermal growth factor 2 positive (HER2+) [4,5]. This subtype is biologically aggressive and carried a dismal prognosis until the arrival of HER2-directed therapy, which has substantially improved curation rates when given as an adjuvant to surgery [5]. HER2-directed therapy also works in most metastatic patients, but only transiently. HER2+ metastatic breast cancer (HER2+ mBC) is highly aggressive at the point when patients develop treatment-refractory disease. HER2 is a member of the epidermal growth factor receptor (EGFR) family with tyrosine kinase catalytic activity and is implicated in a variety of cancers with epithelial origin such as bladder, breast, ovarian, cervical, uterine, prostate, lung, kidney and colorectal cancer [6]. Altered HER2 signaling is a key factor in breast cancer stem cells and is associated with stem cell related pathways, such as the Notch and Wingless/beta-catenin cascades [7]. Through disulfide binding of cysteine-rich extracellular residues, HER2 forms both homodimers and heterodimers with other members of the EGFR family that lead to downstream signaling [8,9]. Upon activation, the downstream signaling cascade initiates the phosphorylation of cytoplasmic tyrosine residues that in turn trigger different signaling pathways such as PLCγ, AKT, MAPK, Src, PKC and PI(3)K [10]. The extensive activation of these signaling pathways orchestrate and promote aberrant cell proliferation, migration, survival and differentiation [4,11]. 

Some breast cancer cells express isoforms of HER2, that are generated through at least two different mechanisms [12,13]. Proteolytic cleavage of HER2 by metalloproteinases was the first mechanism to be discovered [12,14]. The second mechanism involves the alternative initiation of translation from internal methionine codons located at positions 611, 648, 676 or 687 [13,15,16,17,18]. A number of isoforms with varying status of activity have been identified and are collectively referred to as p95HER2 [13,15,19]. The most potent and hyperactive p95HER2 isoform is called 611-HER2-CTF (carboxy-terminal fragment) [7,13,20,21]. This isoform has a short extracellular domain consisting of cysteine residues that are absent in the other isoforms and, due to the lack of a large part of the extracellular domain, forms homodimers more readily compared to HER2 full-length [15,22]. The 611-HER2-CTF isoform is a potent regulator of cancer stem cell features [7]. There is no generally accepted definition of p95HER2 positivity. It has been reported that approximately 80% of HER2 positive patients express a variant of p95HER2, while 20–40% have been defined as p95HER2 positive in research assays [20,22,23]. Patients expressing the hyperactive 611-HER2-CTF isoform of p95HER2 (hereafter known as p95HER2) more frequently develop a metastatic form of breast cancer with a poor prognosis, compared to patients that only express the full-length HER2 receptor [14,15,22,23]. This form of breast cancer is naturally aggressive and has lost the extracellular domain that contains the binding sites for trastuzumab and pertuzumab, leading to therapy resistance [24,25,26,27,28]. p95HER2+ cancers are also largely resistant to small-molecule tyrosine kinase inhibitors [24,25,26,27,28], but may respond to chemotherapy and to lapatinib [29,30]. 

There is currently no available diagnostic assay for identifying p95HER2 positive tumors and no p95HER2-directed therapy. Such an assay would be highly useful for development of personalized medicine, as p95HER2 is an independent prognostic factor and a strong predictive biomarker of response to treatment. Further, p95HER2 represents an attractive therapeutic target, as it is associated with tumor escape and a poor prognosis. Moreover, unlike full length HER2, the p95HER2 variant is highly tumor specific and not expressed in normal tissues. Here, we report the generation and characterization of a high-affinity monoclonal antibody that specifically binds to the hyperactive 611-HER2-CTF isoform of p95HER2, but not to the other known isoforms or to full-length HER2. 

## 2. Materials and Methods

### 2.1. Breast Carcinoma Material

Breast carcinoma biopsy 1 was from a patient in the OsloVal study (REK number 2010/498), where informed consent for research has been obtained. Breast carcinoma biopsies 2 and 3 were from anonymized material with known HER2 status at the Department of Pathology and are used for quality controls and methods development. For biopsy 2 and 3, the HER2 status was known in advance from the routine pathology diagnostic procedures, as performed by validated methods on FFPE material (Ventana 790-4493, clone 4B5; Ventana, AZ, USA) and for sample 2, in addition, situ hybridization using Ventana HER2 dual SISH gene-protein analysis For biopsy 1, HER2 amplification had been shown by previous copy number analysis.

### 2.2. Antibodies

Anti-HER2 (ab214275, Abcam, Cambridge, UK), actin (A2066, Sigma-Aldrich, Oslo, Norway), goat-anti-rabbit-HRP (65–6120, ThermoFisher, Oslo, Norway), goat anti-mouse-AF488 (ab150117, Abcam, Cambridge, UK), goat anti-rat-PE (405406, Biolegend, Oslo, Norway), goat anti-rat-AF546 (A11081, ThermoFisher, Oslo, Norway), goat anti-rabbit-AF647 (A27040, ThermoFisher, Oslo, Norway), horse-anti-mouse-HRP (7076S, Cell Signaling Technology, Danvers, MA, USA) and Fixable Viability Dye eFluor™ 780 (eBioscience, Oslo, Norway).

### 2.3. Animals

The NSG mice were obtained from OUS-Norwegian Radium Hospital, Department of Comparative Medicine. The study was approved by the Norwegian Food Safety Authority and all the procedures were conducted in accordance with guidelines. To establish the p95HER2 orthopedic xenograft mouse model, mice at the age of 6 weeks old were anesthetized with Zoletil (Virbac, Carros, France) and the abdominal area disinfected. A small incision (around 1 cm) was made between nipples 4 and 5, and 5 × 10^5^ p95HER2-T47D cells (C2069, Crown Bioscience, San Diego, CA, USA) in 50 μL PBS injected into the mammary fat pad. The incision was closed by suturing (Polysorb 5-0 Fiolett 75 cm CV11 GL-890, MEDTRONIC NORGE AS, Fornebu, Norway) and sealed by Vevlim Histoacryl Blue (BRA41050044, B. Braun, Melsungen, Germany) and Temgesic (0.3 mg Buprenorfin) injected subcutaneously to reduce postoperative pain. Mice were given 17β-Estradiol-Estradiol (Sigma-Aldrich, Oslo, Norway) in their drinking water (final concentration 5 mg/L) from 10 days before orthotopic transplantation of p95HER2-T47D cells until the end of the study. MDA-MB-468 (HER2+) and MCF7 cell lines were injected subcutaneously into the flank, and tumor growth measured by caliper.

### 2.4. Cell Culture

p95HER2-T47D (C2069, Crown Bioscience), T47d, MDA-MB231, LnCaP, 22Rv1, NALM6 and SUP-T1 cell lines were cultured in RPMI-1640 (Sigma-Aldrich, Oslo, Norway). MCF7, SK-BR-3, MDA-MB-468, A549, PANC-1, VCaP, Du-145 and HEK-239 cell lines were cultured in DMEM (Sigma-Aldrich, Oslo, Norway). Culture Media were supplemented with 100 U/mL Penicillin-Streptomycin (Sigma-Aldrich, Oslo, Norway) and 10% heat-inactivated fetal bovine serum (FBS) (Sigma-Aldrich, Oslo, Norway). Cell lines were incubated at 37 °C with 5% CO_2_ and 100% humidity.

### 2.5. Retroviral Production and Transduction

The retrovirus production was performed as previously described [31] using pBABEpuro-ERBB2 (#40978) plasmid. To make a stable cell line expressing full-length HER2 (MB-MDA-468 HER2+), 5 × 10^4^ cells were seeded per well (24 well plate) and incubated overnight at 37 °C in 5% CO_2_. The next day, 1 mL virus suspension with polybrene (8 mg/mL, Sigma-Aldrich, Norway) was added per well and spun down (900 g, 60 min). The plate was then incubated at 37 °C in 5% CO_2_. After 48 h, the supernatant was discarded and replaced with fresh medium containing 2 μg/mL puromycin (A11138-03, Gibco, Life Technologies, Carlsbad, CA, USA) to select positive cells containing pBABEpuro-ERBB2. Cells were kept in culture with media containing 2 μg/mL puromycin until reaching 80% confluency, then trypsinized and transferred to a T-25 flask.

### 2.6. Generation of Oslo-2 mAb

p95HER2 polyclonal and monoclonal hybridoma generation:

The hybridomas were generated by Aldevron (Freiburg, Germany) after genetic immunization of rats (https://www.aldevron.com/antibody-discovery; https://genovac.com/solutions, accessed on 19 September 2022). Briefly, 8–12 week old rats were injected intradermally with 10 μg of the immunization vector DNA, fixed to gold particles and applied with a gene gun in a weekly rhythm. The first round of sera was taken 10 days after the fourth genetic immunization on day 31 and titers were analyzed by flow cytometry. Sera diluted in PBS/3% FBS, were tested by flow cytometry using cells transfected with test constructs, expressing 611-CTF-HER2, to screen and select candidate rats for further steps. Next, lymphocytes were isolated from the rats and hybridomas were generated based on standardized techniques. To generate hybridomas, isolated B-cells were fused with Ag8 myeloma cells. Hybridomas were plated in appropriate dilutions and supernatants were collected for further screening by flow cytometry (iQue instrument, Sartorius AG, Gottingen, Germany) and/or ELISA. The selected hybridoma candidates were sub-cloned by limited dilution. Collected monoclonal hybridoma supernatants were used for final screening with flow cytometry and/or ELISA.

Hybridoma sequencing, mAb production and purification:

High-throughput sequencing was performed on a final sub-cloned hybridoma by Absolute Antibody on an HiSeq (Illumina, San Diego, CA, USA) sequencer, using cDNA library generated from total RNA. Variable heavy and variable light domains were identified separately. Sequences were compared with known aberrant (i.e., non-functional) antibody genes that are present in many hybridomas and these genes were removed from the analysis when necessary. 

### 2.7. Immunoblotting

Cells were harvested with RIPA buffer (Thermo Fisher Scientific, Oslo, Norway) containing phosphatase and protease inhibitors (No. 78420 and 78430, respectively, Thermo Fisher Scientific, Oslo, Norway) and sonicated (Bioruptor 300, Diagenode, Belgium) for 5 min. The protein concentration was measured by Pierce™ BCA Protein Assay Kit (Thermo Fisher Scientific, Oslo, Norway) according to the manufacturer’s instructions. Immunoblotting was performed as described previously [32]. Primary antibodies anti-HER2 (rabbit-anti-human, 0.66 μg/mL), β-Actin (rabbit-anti-human, 0.67 μg/mL) and HRP-conjugated antibody (goat-anti-rabbit, 0.33 μg/mL) were used. Blots were visualized by ChemiDoc MP imaging system (Bio-Rad, Oslo, Norway).

### 2.8. Flow Cytometry

Cells were washed, pelleted down and resuspended in 100 μL FACS buffer (containing phosphate-buffered saline (PBS), pH 7, 2% fetal bovine serum and 2 mM EDTA). Poly/monoclonal hybridoma supernatants (1:50 dilution) or Oslo-2 mAb (10 μg/mL) were added and incubated for 30 min at 4 °C in the dark. Cells were washed twice, resuspended in 100 μL FACS buffer containing secondary antibody goat anti-rat-PE (0.26 μg/mL) or goat anti-mouse-AF488 (1.33 μg/mL) and Fixable Viability Dye eFluor™ 780 and incubated for 30 min in dark at 4 °C. Cells were washed, resuspended in 200 μL FACS buffer and analyzed on a LSR II flow cytometer (BD Biosciences, Franklin Lakes, NJ, USA). Data were analyzed with FlowJo software (v10.7.1, FlowJo LLC, Ashland, OR, USA).

### 2.9. Immunofluorescence Staining and Fluorescence Confocal Microscopy

For the detection of HER2 and p95HER2 on cell lines, cells were cultured on Chamber Slide (Thermo Fisher Scientific, Oslo, Norway). The cells were fixed with 4% formaldehyde (Thermo Fisher Scientific, Oslo, Norway) for 15 min at 4 °C in the dark and washed twice with PBS. Cells were then permeabilized with 0.1% Saponin (Sigma-Aldrich, Oslo, Norway) for 5 min at room temperature (RT), washed two times for 5 min with PBS and blocked with 10% goat serum (containing 0.1% Saponin) for 60 min at RT. The cells were then incubated with poly/monoclonal hybridomas supernatant (1:100), anti-HER2 (2.65 μg/mL) and Oslo-2 mAb (5 μg/mL) in PBS containing 1% goat serum and 0.1% Saponin for 60 min at RT. After washing three times for 5 min with PBS + Saponin (0.05% Saponin) the cells were incubated with the secondary antibodies goat anti-rat-Alexa 546 (1 μg/mL), goat anti-mouse-AF488 (1.33 μg/mL) and goat anti-rabbit-AF647 (1 μg/mL) in PBS containing 1% goat serum and 0.1% Saponin for 60 min at RT. Cells were washed three times with PBS + Saponin and mounted with ProLong™ Diamond Antifade Mountant with DAPI (Thermo Fisher Scientific, Oslo, Norway). To detect HER2 and p95HER on fresh frozen (FF) murine tissues and xenograft tumors, the same protocol was followed as stated above with an additional 30 min incubation to air-dry the cryosections before fixation. Fluorescence signals were examined on the same day of staining by LSM 880 AiryScan (Carl Zeiss, Jena, Germany). The sensitivity and specificity of the method were evaluated by staining MDA-MB-468 (HER2+) and MCF7 (HER22212) xenograft tumors.

### 2.10. Immunohistochemistry

Staining was performed on fresh frozen (FF) human tissues and xenograft tumors (cryosections, 4 µm). Air-dried sections were fixed with 4% formaldehyde for 5 min at RT and washed for 5 min three times with PBS. Sections were blocked for endogenous peroxidase for 10 min at RT using REAL Peroxidase-Blocking Solution (S2023, Dako, Oslo, Norway) and washed three times for 5 min with dH_2_O. Sections were then incubated with blocking solution (TBS containing 10% goat serum, 5% BSA and glycine at 0.3 M final concentration) for 60 min. Slides were drained and incubated with primary antibodies anti-HER2 (2.65 μg/mL) or Oslo-2 mAb (5 μg/mL) diluted in protein blocking solution for 60 min at RT. Sections were washed three times for 5 min with TBST. Polink-2 Plus HRP Broad DAB detection Kit (D41-18, Golden Bridge International, Bothell, CA, USA) was used according to the manufacturer’s instructions to detect HER2 or p95HER2 target proteins.

The scoring of HER2 protein expression in the present study into 0 (negative), 1+ (weak), 2+ (moderate), and 3+ (strong) was performed according to international standard guidelines, as described in the Ventana anti-HER2/neu (4B5) interpretation guide (Ventana, AZ, USA).

### 2.11. Surface Plasmon Resonance (SPR)

SPR was performed as described previously [33]. In brief, anti-HER2 (5 μg/mL) as the reference and Oslo-2 mAb (5 μg/mL) were covalently immobilized onto the surface of two different flow cells on sensor chip CM5 (2104988, GE Healthcare, Chicago, IL, USA) using Amine Coupling Kit (BR-1000-50, GE Healthcare) and HBS-EP+ buffer. The extracellular domain of p95HER2 peptide with a poly-histidine-tag in the C-terminus (MPIWKFPDEEGACQPCPINCTHSCVDLDDKGCPAEQRASPLTHHHHHH, synthesized by GenScript, Piscataway, NJ, USA) was used as an analyte with serial dilutions from 0.6 to 2500 nM. Kinetics of molecular interaction were processed by global curve fitting to the 1:1 bimolecular interaction model. Biacore T200 (GE Healthcare, Chicago, IL, USA) was used to perform the experiment and all procedures were conducted at 25 °C. Analysis and data quality control were performed automatically by Biacore T200 Evaluation software 3.1. In brief, the binding capacity of the surface (*Rmax*) is calculated as $Rmax=\frac{\mathrm{Oslo}-2\;\mathrm{mAb}}{p95\mathrm{HER}2\;ECD\;\mathrm{peptid}}\;$* R_L_ * S_m_, where *R_L_* is the immobilization level and *S_m_* is the stoichiometric ratio. The equilibrium dissociation constant (*KD*) is calculated as $\mathrm{KD}=\frac{\mathrm{koff}\;(\mathrm{kd})}{\mathrm{kon}\;(\mathrm{ka})\;}$, where *ka* and *kd* are the association and dissociation rates, respectively. 

### 2.12. Epitope Mapping

Overlapping peptides (15 mers overlapping by 4 amino acids) covering the entire p95HER2 extracellular domain and peptides with N-terminal truncations or AA substitutions for the shorter 20 mer epitope (peptide seq: “GVKPDLSYMPIWKFPDEEGA”) were generated and immobilized on a cellulose membrane (PepSpots, JPT Peptide Technologies GmbH, Berlin, Germany). The membrane was rinsed with methanol for 5 min at RT and washed three times for 3 min with TBST. The membrane was blocked by incubating with 5% nonfat skim milk (Bio-Rad, Oslo, Norway) for two hours at RT and then incubated with the Oslo-2 mAb (5 μg/mL) diluted in the same blocking solution for three hours at RT. After washing three times for 5 min in TBST, the membrane was incubated with an HRP-conjugated secondary antibody (horse-anti-mouse, 1.33 μg/mL) diluted in the same blocking solution for two hours at RT. The membrane was washed in TBST and incubated with SuperSignal West Pico chemiluminescent scent substrate (Thermo Fisher Scientific, Oslo, Norway) for 1 min. The membrane was washed repeatedly and gently with TBST and visualized by iBright FL1500 (Invitrogen, Waltham, MA, USA).

### 2.13. Hydrogen Deuterium Exchange (HDX)

Before the labeling reaction, the Oslo-2 mAB was dialyzed in equilibration buffer (10 mM HEPES pH 7.3, 100 mM NaCl) and brought to a final concentration of 6 µM. The antibody antigen complex was prepared at a 1:3 molar ratio (Oslo-2:p95HER2).

The labeling reaction was done by diluting either the Oslo-2 mAb or the (Oslo-2:p95HER2) complex, 20 times in the labeling buffer (10 mM HEPES pD 7.3, 100 mM NaCl), resulting in a final 95% D_2_O labeling buffer. The labeling reaction took place at room temperature and was quenched at four time points (1, 10, 100 and 1000 min) by mixing 50 μL of the reaction with equal volume of ice-cold quenching buffer (Formic Acid 0.8%, 4 M Guanidine hydrochloride, 0.5 M TCEP; final pH 2.3). The quenched samples were stored at −80 °C before proteolysis and liquid chromatography–mass spectrometry steps. The reported experiment consisted of three replicates prepared independently.

### 2.14. Liquid Chromatography and Mass Spectrometry

The quenched samples were thawed on ice and injected on a nanoACQUITY UPLC system with HDX technology (Waters). The temperature of the chamber containing the sample loop as well as the analytical and trap columns was set at 0.5 °C. The temperature of the pepsin column compartment was set at 20 °C. The quenched samples (0.15 pmol/µL) were injected into a 50 µL sample loop and run in a trapping mode, where the protein was passed through a pepsin column (Waters Enzymate 2.1 × 30 mm, 5 μm) and the proteolyzed sample was immediately directed to a trap column (Waters Acquity Vanguard BEH C18, 1.7 µm, 2.1 × 5 mm) to desalt peptide fragments. The flow rate was set to 70 μL/min during the first minute, followed by 100 μL/min for another 2 min with buffer A (0.2% formic Acid, 0.025% Trifluoroacetic Acid pH 2.5). After desalting, the peptides were separated by C18 analytical column (Waters Acquity BEH C18, 1.7 µm, 1.0 × 100 mm) with a linear 5–50% acetonitrile gradient using buffer B (99.9% acetonitrile, 0.1% formic acid and 0.01 % Trifluoroacetic Acid pH 2.5). The elution gradient was run at 40 µL/ min for 17 min. The output of the analytical column was directed to a mass spectrometer (Q-TOF SYNAPT G2-Si, Waters) for peptide identification and determination of the deuterium uptake. The mass spectrometer was operated in the positive ion electrospray mode, with the ion mobility function to minimize spectral overlap using the MS^E^ acquisition mode (Waters Corporation, Milford, MA, USA). Lock mass correction with the Leu-ENK peptide was used to ensure mass accuracy determination. 

### 2.15. HDX-MS Data Analysis

A library of non-deuterated peptides was created using the ProteinLynx Global server 3.0 (PLGS) (Waters) using the following requirements: (1) a mass error for the peptide is below 10 ppm for the precursor ion; (2) theFF peptide has at least two fragmentation products. The level of deuteration in the peptides was determined with DynamX 3.0 (Waters). A manual inspection of all the assignments was conducted to confirm the data or discard the noisy or overlapping spectra.

The reported percentage of deuteration in the HDX differences mapped on the Oslo-2 mAb was calculated by normalization with respect to the maximal theoretical deuterium, which was established as 100%. The maximal theoretical uptake was calculated using the equation:

Max uptake = *N* − *P* − 2

where *N* is the number of amino acids in the peptide, and *P* is the number of prolines in the peptide. This is because prolines in the protein main chain do not have exchangeable hydrogen and the labels in the first two amino acids of the peptide are lost in back exchange.

($\%D=D*\frac{100}{max\;uptake}$) where *D* is the amount of the deuterium for a peptide at a specific time point and “max uptake” is the theoretical maximal amount of deuterium that can be incorporated by a given peptide.

### 2.16. Modeling of p95HER2 Protein and Oslo-2 mAb

The extracellular domain of p95HER2 was modeled using AlphaFold 2.0 [34]. The antibody structure was modeled using the standalone version of AbodyBuilder [35]. The 3D structures of HER2 and p95HER2 were visualized using PyMOL Molecular Graphics System (Version 2.2, Schrödinger, LLC, New York, NY, USA).

### 2.17. Computational Docking

We have used three docking methods, namely, Zdock 3.0.2 [36], Haddock 2.4 [37] and ClusPro 2.0 [38] to predict the top 10 docking poses. The final complex structure was selected based on the consensus of these three methods and calculated based on percentage epitope overlap of each structure with reference to shorter epitope and longer epitope. The docked structures were first filtered based on the highest count of structures with ≥40% epitope overlap and the final consensus structure was selected among the remaining top docked structures based on the highest value of average % of epitope overlap (for pairs having more than 40% epitope overlap). The selection criteria were applied independently to both percentage epitope overlap values (with reference to short and long epitope) and the final structure was selected based on overall performance. 

## 3. Results

### 3.1. Murine Immunization and Hybridoma Screening

**Figure 1:**
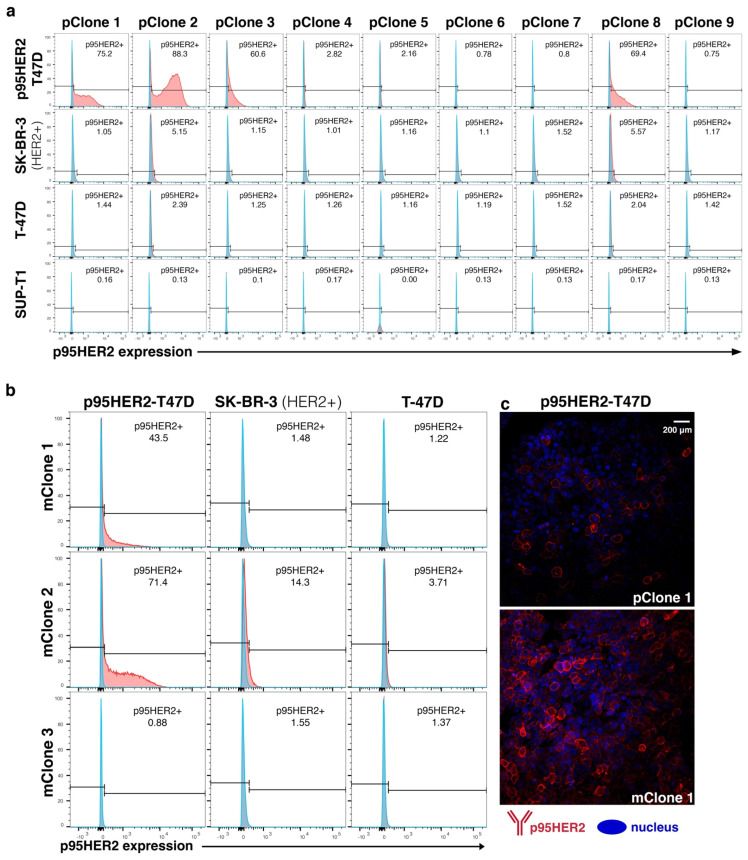
p95HER2 poly and monoclonal hybridoma selection. (**a**) The top nine polyclonal hybridomas from the initial iQue screening were tested by flow cytometry for binding to breast cancer target cells expressing 611-p95HER2 (p95HER2−-T47D) or full-length HER2 (SK-BR-3), and HER2 negative controls including wild type T-47D and SUP-T1 (blue: secondary antibody alone and red: secondary + primary antibodies). pClone 2 had the strongest p95HER2-reactivity, followed by pClones 1, 3 and 8. pClones 2 and 8 were weekly reactive to SK-BR-3 (HER2+), while pClone 1 and 3 had no detectable reactivity against SK-BR-3. None of the pClones were reactive to the HER2 negative target cells (T-47D or SUP-T1). (**b**) Flow cytometry screening of supernatants from monoclonal hybridomas (mClones) generated from pClones 1, 2 and 3. mClone 1 stained p95HER-T47D, and not SK-BR-3. mClone 2 stained both p95HER-T47D and SK-BR-3, while mClone 3 stained neither. (**c**) The same batch of p95HER-T47D cells was stained for immunofluorescence with supernatants from pClone 1 and mClone 1. These results indicate that the reactivity of Clone 1 to p95HER2 was increased from the poly- to monoclonal level.

The generation of p95HER2-specific Abs was performed by immunizing rats with a vector encoding 611-HER2-CTF. To this aim, candidate vectors were first evaluated by transfecting HEK-293 cells with different 611-HER2-CTF constructs and the surface expression of p95HER2 was measured using an anti-tag antibody, and an irrelevant anti-tag antibody as a control (Supplementary Figure S1). Vector pB1-611-CTF-hum.ECD was chosen and injected intradermally with a gene gun. The initial screening of polyclonal hybridoma culture supernatants (HCS) against p95HER2 was performed using the Intellicyt iQue flow cytometry platform. We selected the top nine positive clones based on mean fluorescence intensity (MFI) values (Supplementary Figure S7). The HCS from these nine polyclonal hybridomas (pClones) were then tested by flow cytometry for binding to the cell lines p95HER2-T47D, SK-BR-3, T-47D and SUP-T1. We found that HCS from pClones 1, 2, 3 and 8 bound to p95HER2-T47D but not T47D. Only HCS from pClones 2 and 8 showed any binding to SK-BR-3, a cell line that expresses full-length HER2 (Figure 1a). To confirm these results, immunofluorescence (IF) staining was performed on p95HER2-T47D, SK-BR-3 and T-47D. Here, we found that HCS from pClones 1, 2 and 3 stained p95HER2-T47D, but not T-47D or SK-BR3 (Supplementary Figure S2). 

### 3.2. Subcloning and Evaluation of Monoclonal Hybridoma

Based on these data, we chose pClones 1, 2 and 3 for subcloning into monoclonal cultures (mClones) through limited dilution series. The Intellicyt iQue screening of mClones 1, 2 and 3 demonstrated that only mClone 1 bound specifically to transfected cells, while mClone 2 bound non-specifically to non-transfected cells and mClone 3 was negative (Supplementary Figure S8). To further test all three mClones, we performed flow cytometry analysis on the cell lines p95HER2-T47D, SK-BR-3 and T-47D using the HCS. The flow cytometry results confirmed the iQue screening data (Figure 1b). Only mClone 1 bound specifically to p95HER2-T47D, while mClone 2 bound to both p95HER2-T47D and SK-BR-3. Furthermore, mClone 1 demonstrated stronger reactivity to p95HER2 compared to pClone 1 when assessed by IF (Figure 1c). Based on these screening results, mClone 1 was selected for generating a mAb.

### 3.3. Generation of Oslo-2 mAb and Flow Cytometry Evaluation Using Cell Line Panel

**Figure 2:**
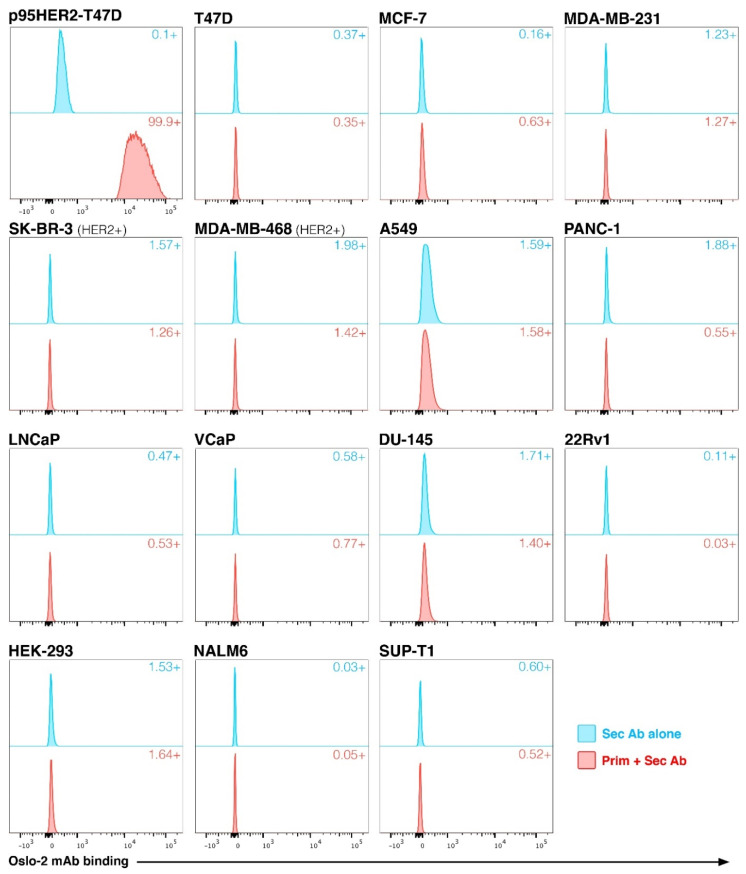
Reactivity and specificity of Oslo-2 mAb against p95HER2. The reactivity of the Oslo-2 mAb was tested by flow cytometry using a panel of 15 HER2+/− cell lines. Oslo-2 was only reactive to the p95HER2-T47D cell line and not to the cell lines expressing full-length HER2 (HER2+ SK-BR-3, MDA-MB-468 and A549), or the HER2 negative breast cancer cell lines T-47D, MCF-7 and MDA-MB-231. The Oslo-2 mAb was also not reactive to any of other malignant cell lines tested.

We then identified the Ab-coding sequences of the mClone 1 hybridoma and generated a synthetic mAb, termed Oslo-2, incorporating these sequences. The reactivity and specificity of the Oslo-2 mAb was evaluated by flow cytometry, using a panel of HER2+/− cell lines originating from different solid tumors or hematological malignancies (Figure 2). In tests with breast cancer cell lines, the Oslo-2 mAb had a strong reactivity to p95HER2-T47D but did not bind to the HER2 positive SK-BR-3 and MDA-MB-468 lines, or to the HER2 negative T47D, MCF7 and MDA-MB-231 cell lines (Figure 2). Moreover, the Oslo-2 mAb did not bind to the HER2+ lung cancer cell line A549, or to HER2 negative prostate cancer, pancreatic cancer, lymphoma and leukemia cell lines (Figure 2).

HER2 and p95HER2 expression were confirmed in eight cell lines by western blot using an antibody against the cytoplasmic domain of HER2 (cytopla-HER2). The results showed that the HER2+ SK-BR-3, MDA-MB-468 (transduced with HER2) and A549 cell lines were positive for HER2, but not for p95HER2 (Supplementary Figure S3). Only the p95HER2-T47D cell line was positive for p95HER2 (Supplementary Figure S3). Taken together, the results demonstrate that the Oslo-2 mAb binds specifically to p95HER2, does not bind full-length HER2 and does not exhibit cross-reactivity to other antigens in any of the cell lines tested.

### 3.4. Immunofluorescence and Immunohistochemistry Evaluation of Oslo-2 mAb

**Figure 3:**
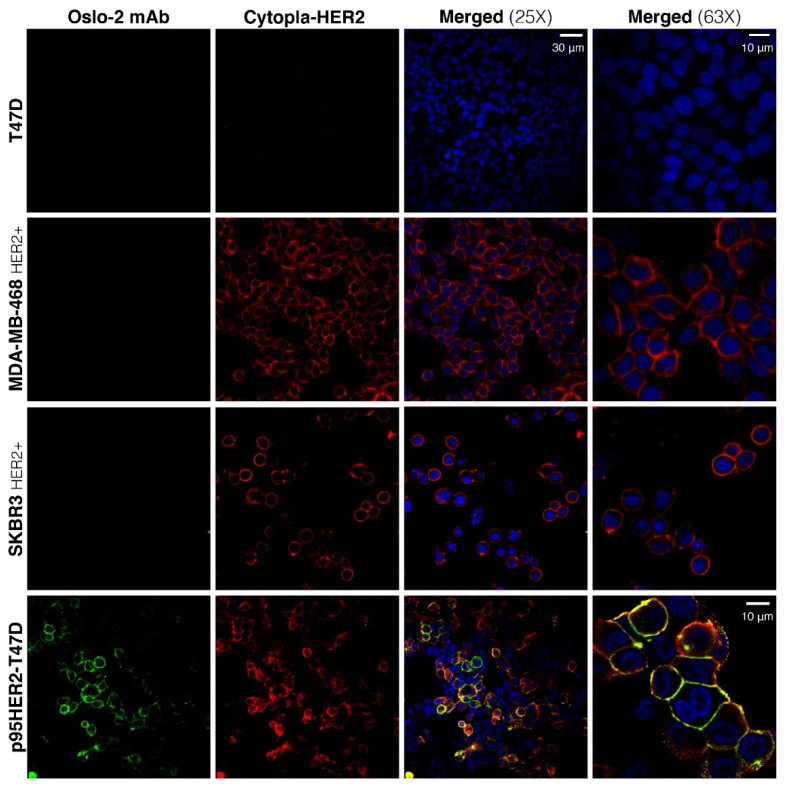
Immunofluorescent staining of breast cancer cell lines using Oslo-2 mAb. Among different breast cancer cell lines, Oslo-2 mAb was only reactive to the p95HER2-T47D cell line and did not show any reactivity to HER2+ SK-BR-3 and MDA-MB-468, while Cytopla-HER2 stained positively p95HER2-T47D and HER2+ SK-BR-3 and MDA-MB-468 cell lines. Neither Oslo-2 mAb nor Cytoplasm-HER2 was reactive to HER2- T47D. Oslo-2 mAb signals were overlapped with Cytopla-HER2 signals, confirming the specific binding of Oslo-2 mAb to p95HER2.

The properties of the Oslo-2 mAb were further evaluated by IF and immunohistochemistry (IHC) on formalin-fixed cells and on fresh-frozen (FF) xenograft tumors and tissue specimens. We performed double-IF staining with both the Oslo-2 mAb and the cytopla-HER2 mAb on formalin-fixed breast cancer cell lines grown on chamber slides. The cytopla-HER2 mAb served as a co-localization reference, to indicate that the Oslo-2 mAb binds p95HER2 and is not a non-relevant target. As expected, the cytopla-HER2 Ab positively stained SK-BR-3, MDA-MB-468 (HER2+) and p95HER2-T47D, but was negative for T47D (Figure 3). The Oslo-2 mAb exhibited strong reactivity only to p95HER2-T47D and no reactivity to any of the other cell lines, including those expressing full-length HER2. The Oslo-2 mAb staining co-localized with that of the cytopla-HER2 Ab, consistent with a specific staining of p95HER2 (Figure 3). Both the Oslo-2 and the cytopla-HER2 stainings depicted p95HER2 expression (in p95HER2-T47D) mainly in the membrane, with a low-level expression in the cytoplasm and nucleus.

**Figure 4:**
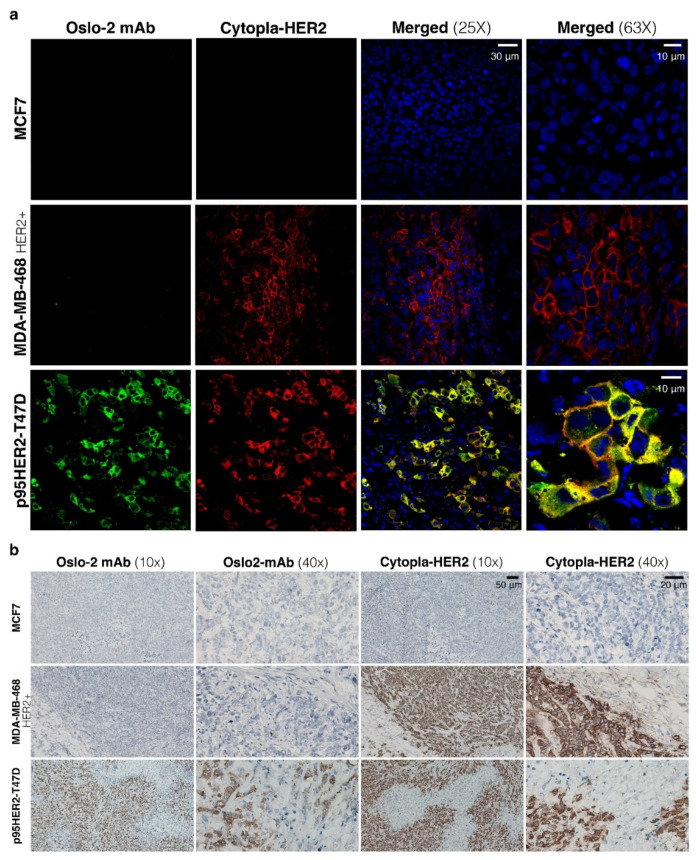
Immunofluorescence and immunohistochemistry staining of breast cancer xenograft using Oslo-2 mAb. (**a**) IF staining of three breast cancer xenograft tumors showed that both Oslo-2 mAb and Cytoplasmic-HER2 mAb positively stained p95HER2-T47D xenograft tumor and signals was colocalized. MDA-MB-468 (HER2+) was only reactive to Cytopla-HER2 mAb, but MCF7 (HER2-) was negatively stained with both antibodies. (**b**) IHC staining of the same xenograft tumor samples confirmed results that were obtained by IF, where Oslo-2 mAb was only reactive to p95HER2-T47D xenograft tumor and Cytopla-HER2 mAb positively stained both p95HER2-T47D and MDA-MB-468 (HER2+) xenograft tumors (both IF and IHC were performed on serial sections).

Next, IF and IHC were performed on p95HER2-T47D, MCF7 (HER2-) and MDA-MB-468 (HER2+) FF xenograft tumors. The cytopla-HER2 Ab positively stained both p95HER2-T47D and MDA-MB-468 (HER2+), but the Oslo-2 mAb only recognized p95HER2-T47D (Figure 4a). MCF7 was negative with both antibodies. IHC staining of the same FF xenograft tumors confirmed the IF data, where Oslo-2 mAb was specifically reactive to p95HER2-T47D, but not to HER+/− xenograft breast cancer tumors (Figure 4b). Moreover, the Oslo-2 mAb was not reactive to normal mouse tissues such as heart, lung, kidney, muscle and liver (Supplementary Figure S4).

**Figure 5:**
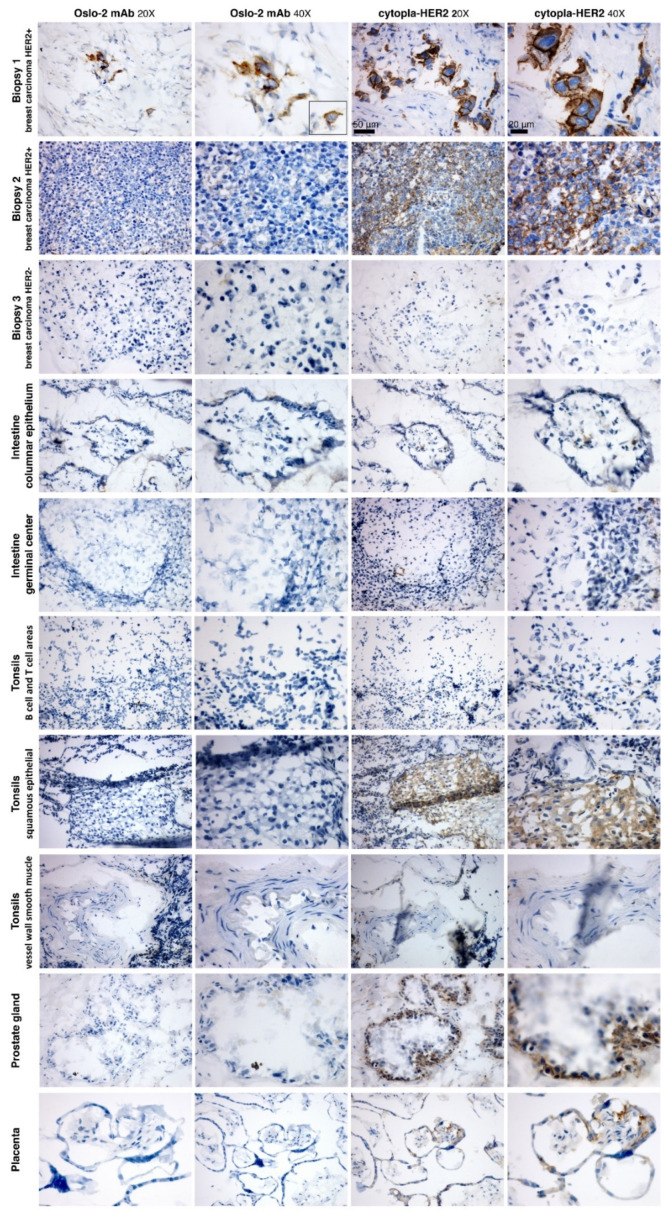
Immunohistochemistry staining of breast cancer and normal tissues. Human breast cancer biopsies (top 3 rows) and a panel of human normal tissues (bottom 7 rows) were stained for IHC with the Oslo-2 (p95HER2) and cytopla-HER2 antibodies. Only biopsy 1 stained positive with Oslo-2 mAb. Biopsy 1 and 2 stained positive with cytopla-HER2. Biopsy 3 (HER2 negative ductal breast carcinoma) stained negative for both cytopla-HER2 and Oslo-2 mAb. Normal tissue panel: columnar epithelium and lymphoid germinal center in the intestine, tonsil vascular smooth muscle and B cell and T cell areas in tonsils were negatively stained with both antibodies. Squamous epithelium in tonsils, prostate glands and trophoblastic cells in the placenta were weakly positive for cytopla-HER2, but negative for Oslo-2 mAb. No non-specific or background staining was observed in IHC for the Oslo-2 mAb. Biopsy 1 Oslo-2 mAb 40× image contains a box that is cut out from a location which is in the 20× image field of view, but outside of the field of view for the 40× image.

To investigate whether the Oslo-2 mAb was reactive to human HER2+/− normal tissues and human HER2+/− breast cancer tumors, IHC was performed using the Oslo-2 and cytopla-HER2 antibodies. The panel consisted of three breast carcinoma biopsies (strongly HER2+, moderately HER2+, HER2 negative), as well as normal tonsils, intestine, prostate and placenta tissues samples. IHC showed that the Oslo-2 mAb was reactive to breast cancer biopsy 1, but not biopsy 2 (Figure 5). Breast cancer sample 1 and 2 stained positive with the cytopla-HER2 antibody, while biopsy 3 stained negative with both antibodies. The IHC results for biopsies 2 and 3 by the cytopla-HER2 antibody (Figure 5) corresponded well with the previously registered HER2 IHC status (2+ and 0, respectively), as recorded from assessments by standard, validated methods on FFPE samples from the same patients. Squamous epithelium in tonsils, prostate glands and trophoblastic cells in the placenta stained weakly positive for cytopla-HER2, but negative with the Oslo-2 mAb. The columnar epithelium and lymphoid germinal center in the intestine, the vascular smooth muscle and the B cell and T cell areas in tonsils were negative for both Oslo-2 and cytopla-HER2 (Figure 5). There was no evidence of unspecific staining or cross-reactivity for the Oslo-2 mAb.

### 3.5. Oslo-2 mAb Affinity and Epitope Mapping

**Figure 6:**
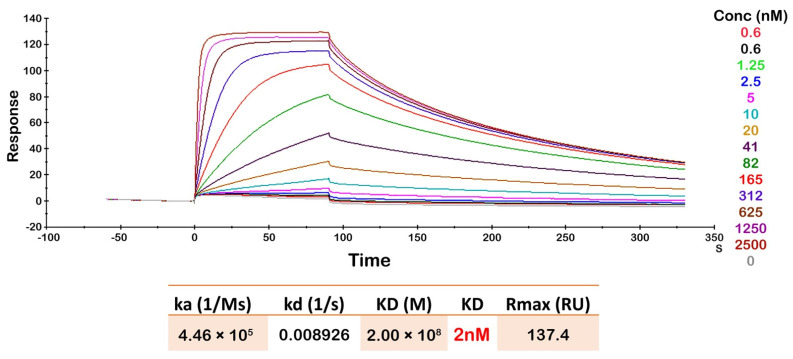
Surface plasmon resonance analysis of Oslo-2 mAb binding affinity: A peptide representing the extracellular domain of p95HER2 was used as an analyte. with serial dilutions from 0.6 to 2500 nM, to determine Oslo-2 mAb binding affinity. Cytopla-HER2 mAb was applied as a reference. The Oslo-2 and cytopla-HER2 mAb were covalently immobilized onto the surface of two different flow cells on a sensor chip. The Oslo-2 mAb data were corrected by subtracting the signal from the control cytopla-HER2 mAb. As shown, the association *(ka)* rate increased with increasing p95HER2 peptide concentration. Bimolecular interaction model 1:1 showed a low equilibrium dissociation constant (*KD* = 2 nM) for Oslo-2 mAb with a high maximal binding response (*Rmax*) at 137 RU. The data shown are representative from two independent experiments.

SPR analysis was performed to determine the binding affinity of Oslo-2 mAb to the p95HER2 peptide epitope (Figure 6). As expected, the association (*ka*) rate increased with increasing p95HER2 peptide concentration. The Oslo-2 mAb had a low equilibrium dissociation constant (*KD* = 2 nM) with p95HER2 peptide, representing a high affinity interaction with the maximal binding response (*Rmax*) at 137 RU (Figure 6).

**Figure 7:**
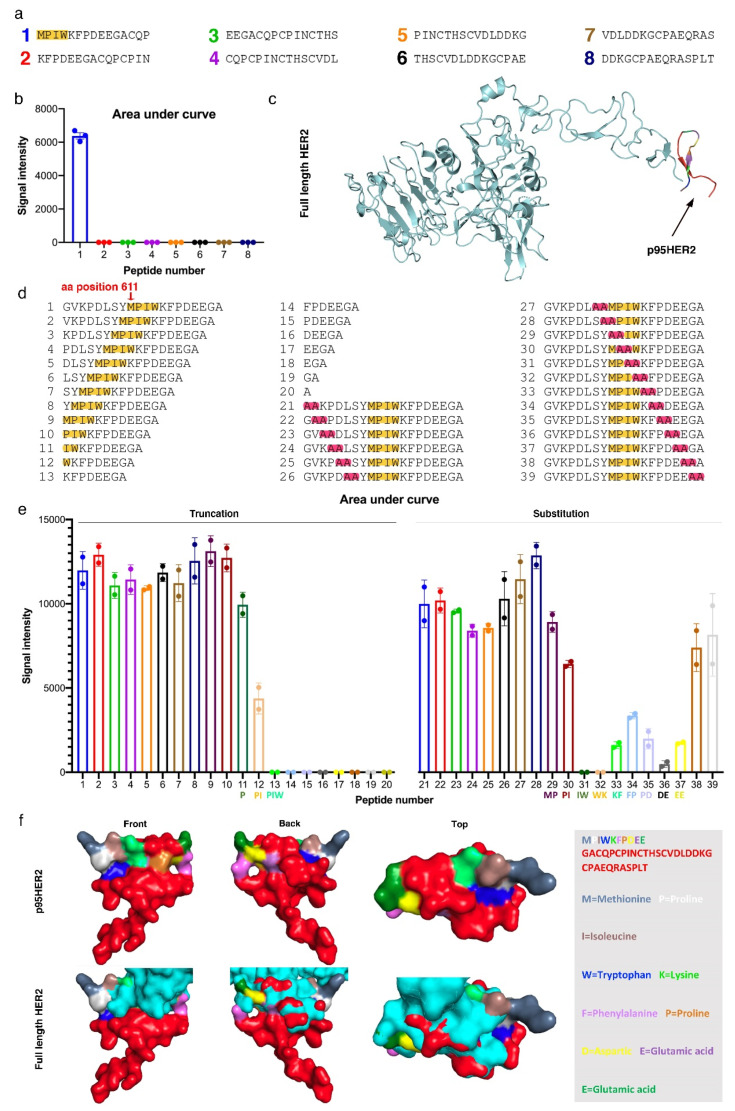
Oslo-2 mAb epitope mapping. (**a**) Serial overlapping peptides covering the p95HER2 extracellular domain, with 11 aa overlap between consecutive peptides. (**b**) Signal intensity representing binding of Oslo-2 mAb to respective peptides, showing that Oslo-2 mAb was reactive only to peptide 1, containing “MPIW”. The assay was performed in triplicates. (**c**) 3D structure of extracellular part of full-length HER2, with p95HER2 region highlighted. (**d**) Peptides 1–20: Truncated peptides covering 633–622 in full-length HER2, truncated from N-terminal. Peptides 21–39: Two amino acid alanine substitutions in peptides covering 633–622 in full-length HER2. (**e**) Signal intensity representing binding of Oslo-2 mAb to respective peptides (duplicates). Truncated peptide data shows that sequence PIW is crucial for binding. Alanine substitution data reveals that KFPDEE is also required. (**f**) 3D structure of full-length HER2 and p95HER2, depicting that the Oslo-2 mAb binding epitope, PIWKFPDEE, is continuous and hidden in full-length HER2. The sequence and color coding for the p95HER2 are shown on the right.

To identify the specific epitope recognized by Oslo-2 mAb, we performed epitope mapping using synthetic overlapping peptides covering the p95HER2 extracellular domain. Sequential consecutive 15mer peptides overlapping by 11 amino acids were generated (Figure 7a) and immobilized on cellulose membranes. The results showed that the Oslo-2 mAb only binds to peptide 1 (Figure 7b), which suggested that the 4 amino acid sequence MPIW was essential for binding. This sequence corresponds to positions 611-614 in full-length HER2.

We next investigated if any additional amino acids were involved in Oslo-2 mAb binding and if the binding epitope was continuous (epitopes where the key binding residues are located in a linear sequence) or discontinuous (epitopes that are composed of two or more binding regions which are found adjacent only in the tertiary protein structure). To this end, we selected region 603–622 in HER2, that spans the N terminus of the p95HER2 extracellular domain and contains the MPIW epitope. We generated peptides that contained either N-terminal truncations for this sequence or sequential peptides that substituted two alanine residues at each position (Figure 7d). N-terminal truncations suggested that methionine-611 does not play a key role in the Oslo-2 mAb binding, as the first reduction of binding was observed after the loss of proline-612. Binding was further decreased with the deletion of isoleucine-613 and lost when tryptophan-614 was removed (Figure 7e). The results from double-alanine substitutions further revealed that substituting amino acids downstream of the MPIW epitope also affected binding, mainly from position 619–620 (Figure 7e). Therefore, we conclude that the binding epitope of Oslo-2 mAb is continuous and requires the sequence “PIWKFPDEE”, corresponding to position 612–620 in HER2 (Figure 7f).

### 3.6. Oslo-2 mAb Paratope Mapping by Hydrogen Deuterium Exchange Mass Spectrometry

Hydrogen-deuterium exchange (HDX) is an experimental approach used to obtain structural and dynamic information about proteins. The method measures the exchange of amide protons from the peptide bonds of a protein with deuterons from the solvent [39]. HDX has been shown to be useful for mapping paratopes and epitopes [40,41]. Here, we measured HDX by mass spectrometry (HDX-MS) to identify regions of the Oslo-2 mAb that exhibit a reduction in HDX upon binding of the antigen p95HER2, thus providing structural and dynamic insight into the antibody–antigen interaction.

**Figure 8:**
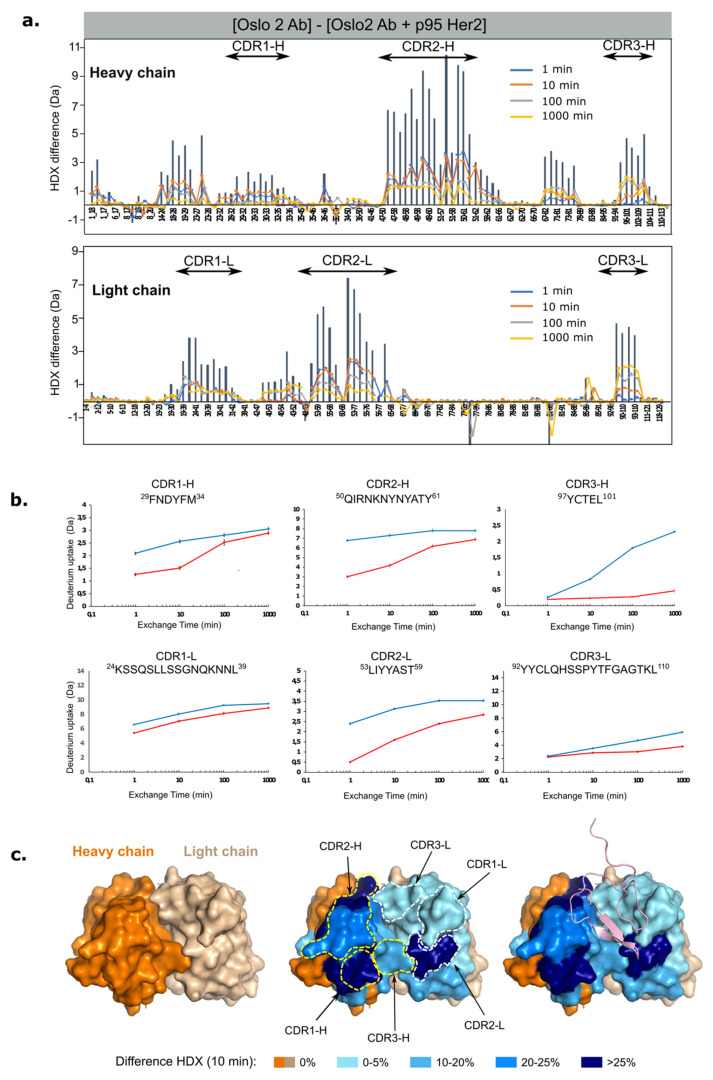
HDX analysis and paratope mapping of the Oslo-2 mAb. (**a**) Butterfly difference plot showing the differences in deuterium uptake (ΔHDX) between Oslo-2 mAb and the (Oslo-2 mAb:p95HER2) complex. Each bar corresponds to a unique Oslo-2 mAb peptide from the peptide library (ordered from N- to C-terminus based on the first amino acid of each peptide; first and last amino acid in peptide are indicated below bars). The Y axis represents the difference in D uptake for a given peptide. The differences in deuterium uptake are shown for each peptide at each labeling time point: blue—1 min; orange—10 min; gray—100 min; yellow—1000 min. The height of the vertical gray bars represents the cumulative magnitude of the HDX difference, which is the sum of the mass differences between Oslo-2 mAb and the (Oslo-2 mAb:p95HER2) complex at each time point. The positive values represent regions of the antibody that are more structured and/or less solvent accessible when the antigen is bound. (**b**) The deuterium uptake plots for representative peptides of the CDR regions in the heavy and light chain. The red traces show the uptake for the (Oslo-2 mAb-p95HER2) complex and the blue traces for the Oslo-2 mAb in the unbound form. (**c**) Top view of the computational model of the Oslo-2 mAb represented as a surface. (Left) Heavy chain in dark orange and light chain in pale orange. (Middle) The HDX differences after 10 min of exchange are mapped in blue on the structural model. Different shades of blue correspond to magnitude of HDX differences observed (legend in figure; for details see Material and Methods). The predicted CDR regions (according to Kabat) on heavy and light chains are circled with yellow and white, respectively. (Right) The structural model of (Oslo-2 mAb:p95HER2) based on molecular docking. The p95HER2 is shown as a pink ribbon.

**Figure 9:**
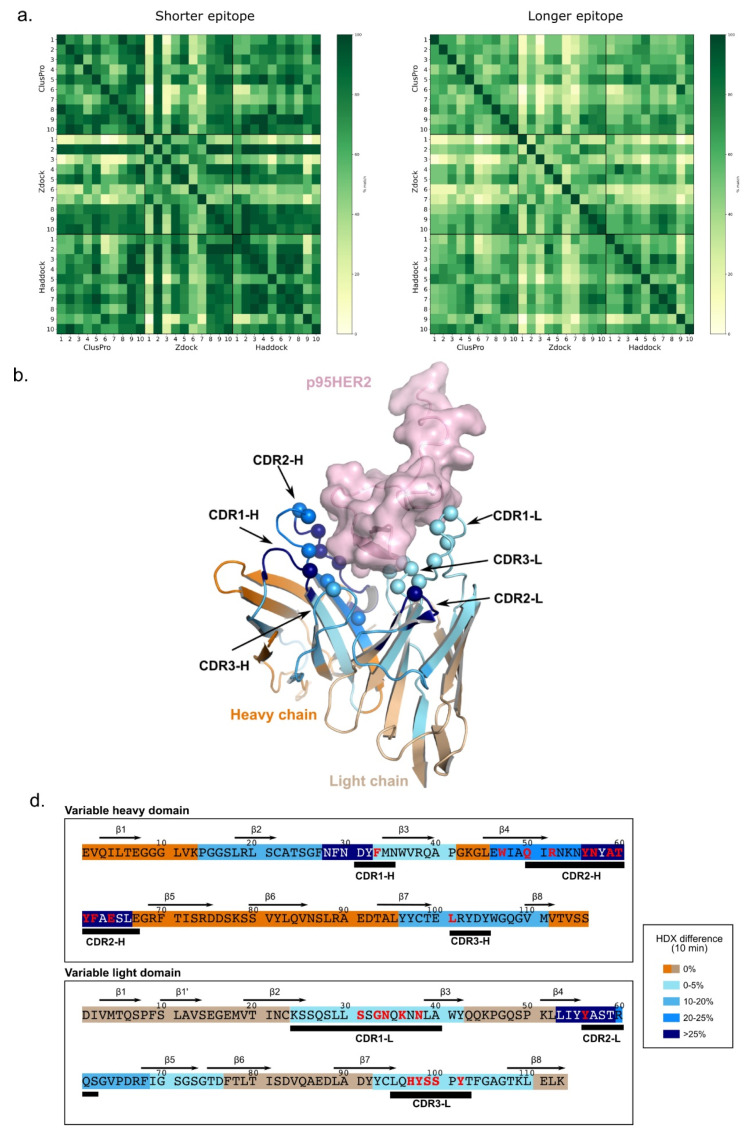
Computational analysis of p95HER2 binding to Oslo-2 mAb. (**a**) A heatmap comparing top 10 models from three different docking methods (ClusPro, Zdock and Haddock) based on percentage epitope overlap with reference to shorter epitope (left) and longer epitope (right). (**b**) The final selected structure of the p95HER2-Oslo-2 antibody complex. p95HER2 is shown as transparent pink surface. Light and heavy chains (variable domains) are shown in ribbon representation and colored based on HDX protection observed at 10 min (see legend in **d**). CDRs are labelled and residues interacting with p95HER2 are shown as spheres. (**c**) The sequence of variable heavy and variable light chains of Oslo-2 Ab colored by difference in HDX at 10 min. Secondary structure elements are indicated on top and position of predicted CDRs according to Kabat is indicated below the sequence. Residues that are interacting with p95HER2 are shown as red letters.

Briefly, the Oslo-2 mAb and the (Oslo-2:p95HER2) complex were each incubated in a deuterated buffer. The reaction was quenched at five time points, and samples were proteolyzed on a pepsin column before mass spectrometric analysis. We focused on identifying peptides in the Oslo-2 mAb that cover the variable regions of the antibody heavy and light chains (Supplementary Figure S5). The differences in HDX for peptides covering identical regions of Oslo-2 mAb in isolation and in complex with p95Her2 were identified and plotted for each of the peptides (Figure 8). We observe strong HDX protection (up to 40% for some peptides) in segments of both the variable heavy and variable light domains at all time points. In each of the chains, the regions with highest protection align very well with the predicted complementarity-determining regions (CDRs) (Figure 8 and Figure 9). The regions flanking the CDRs are also structurally better organized upon binding p95HER2, as reflected in HDX protection. This is most likely due to an allosteric effect that occurs upon binding to p95HER2, rather than direct binding.

The heavy chain shows the strongest protection around CDR1-H and CDR2-H (Figure 8c and Figure 9d; blue intensity correlates with %HDX difference). The protected region around CDR1-H spans residues 18–34 and includes two beta strands (β2 and β3) and a loop between them. The exposed loop (28–34) matches well with the predicted CDR1-H (31–35) and most likely binds directly to p95HER2. As a result, the neighboring beta strands become more rigid. The protected region encompassing CDR2-H spans residues 46–66, which include β4 and a long loop. The CDR2-H predicted by the Kabat method [42,43] extends from position 50 to 68, where the HDX-protected region matches the predicted CDR2-H almost perfectly. Finally, the region that includes CDR3-H comprises residues 96–111, the N-terminal portion of β7 and a long loop that ends with a short β8. In this region, the kinetics of deuterium uptake during the first minute of exchange show no difference in HDX (Figure 8b, upper right panel). However, the differences in deuterium uptake increase with time. These results suggest that the CDR3-H region of the antibody has some degree of structural rigidity even in the absence of p95HER2, resulting in a low level of HDX at early time points. Upon binding of p95HER2, this region becomes even more structurally rigid, leading to the increased HDX differences over time.

HDX differences are generally less pronounced in the light chain, but they are also clearly located in regions containing the predicted CDRs. Here, the protected regions around CDR1-L and CDR -3-L are less protected upon antigen binding than in the region containing CDR2-L (Figure 8 and Figure 9). Interestingly, the peptides in CDR3-L also show an increasing HDX difference with time, similar to that observed around CDR3-H (Figure 8b, far right).

In summary, the HDX-MS experiments agree well with the CDRs predicted by the Kabat method. The regions with the strongest observed differences are clearly located in the loops on the surface of the antibody (Figure 8c and Figure 9). Our experimental data allowed us to map the para-top regions of the Oslo-2 mAb and provided valuable information about the structure and dynamics of the antibody. Using this information, we proceeded to generate a computational atomistic model of the (Oslo-2 mAb: p95HER2) complex using information-driven docking (Figure 8c, right, and Figure 9).

### 3.7. Docking Study of Oslo-2 mAb

The N-terminal region of p95HER2 containing epitope, modeled using AlphaFold 2.0 [34], is in good agreement (RMSD: 0.631 Å) with the crystal structure of HER2 protein (PDB id. 7MN5) (Supplementary Figure S6). However, the C-terminal region is disordered and therefore not available in any crystal structure. We performed an information-driven docking using the region “MPIWKFPDEE” (residue 1–10) of p95HER2 obtained from alanine scanning and the paratope regions of Oslo-2 mAb obtained from the HDX experiments. Although we provided a ten residue long epitope as a binding region, the remaining structure of p95HER2 was not blocked for binding, allowing the paratope to accommodate itself on the whole epitope region. The top ten structures obtained from three docking methods (Zdock 3.0.2 [36], Haddock 2.4 [37] and ClusPro 2.0 [38]) showed a good overlap within the epitope region (Figure 9a). The docking poses from the three different methods were filtered further based on highest epitope overlap to identify the final consensus structure [44]. The percentage epitope overlap was calculated with respect to shorter and longer epitopes to avoid length bias. The consensus structure was selected based on two selection criteria. First, we filtered the top structures based on the count of structures showing more than 40% overlap. Second, the final structure was selected among these top structures based on average percentage of epitope overlap (for structures with more than 40% epitope overlap) (Figure 9b). The computationally predicted epitope and paratope regions for the final selected structure were further mapped on the experimental observations (Figure 9c), showing that CDR2-H contributes more to binding compared to CDR3-H. On the other hand, CDR3-L contributes significantly to binding. The residue-wise interaction for each paratope residue is provided in Supplementary Table S1.

## 4. Discussion

In this study, we have generated and characterized a monoclonal antibody that specifically binds to the hyperactive 611-CTF isoform of p95HER2, but not to the other isoforms or to full-length HER2. The mAb, called Oslo-2, has a high affinity and shows no cross-reactivity against a panel of 14 cell lines from solid and hematological cancer forms. There is also no evidence of cross-reactivity against the human and murine normal tissues included in our panels. The mAb works both for flow cytometry, IHC and IF. As expected, the Oslo-2 mAb IHC staining co-localized with an antibody against the cytoplasmic domain of HER2, mostly in the membrane. We could also detect some Oslo-2 mAb in the cytoplasm and nucleus, as reported previously [13]. The epitope mapping shows that the mAb binds to the N-terminal extracellular region of the truncated 611 p95HER2 fragment and suggests that a consecutive 9mer aa sequence is required for binding. This finding is consistent with the high specificity observed in the functional experiments and suggests that the identified epitope is at least partially masked in full-length HER2, but is exposed in 611-CTF. The epitope overlaps the reported epitope for another p95HER2-binding Ab [45], but includes two additional aa, corresponding to position 619–620 in full length HER2.

Furthermore, we conducted HDX experiments to map the paratope regions of the Oslo-2 mAb antibody. Strong HDX protections due to antigen binding were observed in the predicted CDR regions of the mAb. We found that the dynamics changed more dramatically upon p95HER2 binding of the variable heavy domain, compared to the variable light domain. Further, we identified CDR-1 and CDR-2 on the heavy chain and CDR-2 on the light chain to be the regions that were structurally and dynamically most affected by p95HER2 binding. The epitope and paratope information were further used to computationally predict the (Oslo-2 mAb:p95HER2) complex structure and assessment of interacting residues. The computational analysis predicted the important residues in the paratope and epitope for binding. 

There is currently no commercially available antibody or diagnostic assay for identifying p95HER2 positive tumors. Such an assay would be highly useful for the development of personalized medicine, as p95HER2 is an independent prognostic factor as well as a strong predictive biomarker of response to treatment. Of note, 611-CTF is the only isoform of p95HER that extensively induces expression of genes involved in metastasis and development of malignancy, such as MMP1, ANGPTL4, MET, IL-11, CD44, BCL2A1, ADAM9, PLAUR, EPHA1, EGFR and TGF-α [15]. For clinical decision making, it will be important to screen patients with a mAb that avoids the false positive detection of other p95HER2 isoforms, as well as of full-length HER2. A study from Sperinde et al. suggests that approximately 80% of HER2+ breast cancer cases express the 611-CTF-p95HER2 isoform, 1 to 20-fold higher than negative controls. However, based on the cut-off that they defined, only 30% of the cases could be considered as 611-CTF-p95HER2+ [22]. This issue, with a high number of patient cases suspected to express 611-CTF-p95HER2 at low levels, points to a need for an antibody with a high-affinity to avoid false negative results. Based on our data, the Oslo-2 mAb possesses a high-affinity. The SPR data (*KD* = 2 nM and *Rmax* = 137 R) indicate that the affinity is four-times higher than for a p95HER2 antibody generated by others [20,45]. Taken together, the high affinity and specificity of the Oslo-2 mAb suggest that it may be a reliable tool for diagnosing p95HER2+ breast cancer cases. The Oslo-2 mAb may also be used to investigate the presence and clinical significance of p95HER2+ in other cancer forms known to have HER2+ subgroups, such as glioblastoma and gastric cancer. Currently, there is limited knowledge on this.

The p95HER2 611 isoform represents an attractive therapeutic target that is associated with tumor escape, cancer stem cell properties and a poor prognosis. The Oslo-2 mAb may be used to develop a range of agents, including naked antibody, antibody drug conjugates [46], bispecific T cell engagers [45] or T cells retargeted with chimeric antigen receptors (CAR-T cells). Depending on the chosen antibody tail, an Oslo-2-based Ab may engage Fc receptors on innate immune cells and mediate antibody-dependent cellular toxicity, similar to trastuzumab [47]. The activation of downstream signaling from 611-CTF-p95HER2 is ligand-independent and based on dimerization. The short remaining extracellular domain consists of cysteine-reach residues that facilitate disulfide binding and forms homodimers more readily compared to full-length HER2 [15,22]. The high-affinity Oslo-2 mAb may potentially be used to inhibit signaling from p95HER2-based homodimerization, but this would need to be tested. In the metastatic setting, anti-p95HER2 therapies may address a huge medical need, as metastatic p95HER2+ breast cancer is aggressive, resistant to current therapies and carries a dismal prognosis. The high affinity and specificity of the Oslo-2 mAb makes it a good candidate for targeting even low levels of p95HER2. Unlike full-length HER2, the p95HER2 variant is highly tumor specific and not expressed in normal tissues. This is important as there are concerns over targeting full-length HER2 with cell therapies, bi-specific antibodies and other potent novel therapeutic approaches, due to HER2 expression in normal tissues such as heart and lung. This concern was highlighted by fatal lung toxicity in the first patient treated with HER2-targeting CAR T cell therapy [48]. Humanization of the Oslo-2 mAb may be desirable for avoiding unwanted xeno-reactivity, but may in some cases affect other properties, including the target binding by the scFv used in CAR constructs and some bi-specific antibodies.

The high affinity of the Oslo-2 mAb may be important to prevent low-p95HER2-mediated tumor escape. This property, along with the lack of p95HER2 expression in normal tissues, may allow for developing effective and well-tolerated therapy in the adjuvant setting, i.e., before/after radical surgery. The introduction of trastuzumab against HER2+ breast cancer in the adjuvant setting is regarded as one the most clinically important breakthroughs in cancer therapy, with substantial impact on survival. Before the use of trastuzumab, the HER2+ group carried the worst prognosis within breast cancer, even poorer than the triple negative subgroup [49]. With today’s HER2-directed therapy, the prognosis is good, but less favorable for the p95HER2+ subgroup [7,24]. It is thus possible that adjuvant therapy with a p95HER2-targeting mAb may have a huge medical impact and improve curation rates.

We conclude that the Oslo-2 mAb has a high affinity and binds specifically to the biologically active p95HER2 isoform 611. The mAb may be used for development of diagnostic assays and therapies for p95HER2+ breast cancer, and possibly also other p95HER2+ cancer forms. For these purposes, there will be a need to further characterize the Oslo-2 mAb, including a more comprehensive screening in patient material.

## Data Availability

Data supporting the reported results can be obtained from the corresponding author.

## Supplementary Materials

The following supporting information can be downloaded at: https://www.mdpi.com/article/10.3390/cancers14194859/s1, Figure S1: Expression of 611-CTF-HER2 in cells used for rat immunization and serum screening; Figure S2: Immunofluorescence staining of breast cancer cell lines using polyclonal hybridomas culture supernatants; Figure S3: p95HER2 and HER2 expression in different cell lines; Figure S4: Immunofluorescence staining of mouse tissues using Oslo-2 mAb; Figure S5: Peptide coverage map for HDX experiments; Figure S6: Modeling of p95HER2 structure.; Figure S7: Early-stage polyclonal hybridoma cultures supernatants screening against p95HER2; Figure S8: Early-stage monoclonal hybridoma cultures supernatants screening against p95HER2; Table S1: Interaction of each paratope residue with epitope residues within 4.5 Å contact distance.

## Abbreviations

CAR	chimeric antigen receptor
CDR	complementarity-determining regions
CTF	carboxy-terminal fragment
cytopla-HER2	cytoplasmic domain of HER2
EGFR	epidermal growth factor receptor
FF	fresh-frozen
HDX	hydrogen deuterium exchange
HER2+	human epidermal growth factor 2 positive
IF	immunofluorescence
IHC	immunohistochemistry
mAb	monoclonal Ab
mBC	metastatic breast cancer
mClone	monoclonal hybridoma culture
MS	mass spectrometry
p95HER2	611-CTF p95HER2 isoform
pClone	polyclonal hybridoma culture
scFv	single-chain variable fragment
SPR	surface plasmon resonance
RT	room temperature

## References

[1] Baselga J., Norton L. (2002). Focus on breast cancer. Cancer Cell.

[2] Perou C.M., Sorlie T., Eisen M.B., van de Rijn M., Jeffrey S.S., Rees C.A., Pollack J.R., Ross D.T., Johnsen H., Akslen L.A. (2000). Molecular portraits of human breast tumours. Nature.

[3] Caswell-Jin J.L., Lorenz C., Curtis C. (2020). Molecular Heterogeneity and Evolution in Breast Cancer. Annu. Rev. Cancer Biol..

[4] Owens M.A., Horten B.C., Da Silva M.M. (2004). HER2 amplification ratios by fluorescence in situ hybridization and correlation with immunohistochemistry in a cohort of 6556 breast cancer tissues. Clin. Breast Cancer.

[5] Loibl S., Gianni L. (2017). HER2-positive breast cancer. Lancet.

[6] Yan M., Schwaederle M., Arguello D., Millis S.Z., Gatalica Z., Kurzrock R. (2015). HER2 expression status in diverse cancers: Review of results from 37,992 patients. Cancer Metastasis Rev..

[7] Pupa S.M., Ligorio F., Cancila V., Franceschini A., Tripodo C., Vernieri C., Castagnoli L. (2021). HER2 Signaling and Breast Cancer Stem Cells: The Bridge behind HER2-Positive Breast Cancer Aggressiveness and Therapy Refractoriness. Cancers.

[8] Van Der Geer P., Hunter T., Lindberg R.A. (1994). Receptor protein-tyrosine kinases and their signal transduction pathways. Annu. Rev. Cell Biol..

[9] Schulze W.X., Deng L., Mann M. (2005). Phosphotyrosine interactome of the ErbB-receptor kinase family. Mol. Syst. Biol..

[10] Yarden Y., Sliwkowski M.X. (2001). Untangling the ErbB network. Nat. Rev. Mol. Cell Biol..

[11] Citri A., Yarden Y. (2006). EGF-ERBB signalling: Towards the systems level. Nat. Rev. Mol. Cell Biol..

[12] Christianson T.A., Doherty J.K., Lin Y.J., Ramsey E.E., Holmes R., Keenan E.J., Clinton G.M. (1998). NH2-terminally truncated HER-2/neu protein: Relationship with shedding of the extracellular domain and with prognostic factors in breast cancer. Cancer Res..

[13] Anido J., Scaltriti M., Bech Serra J.J., Josefat B.S., Rojo Todo F., Baselga J., Arribas J. (2006). Biosynthesis of tumorigenic HER2 C-terminal fragments by alternative initiation of translation. EMBO J..

[14] Xia W., Liu L.H., Ho P., Spector N.L. (2004). Truncated ErbB2 receptor (p95ErbB2) is regulated by heregulin through heterodimer formation with ErbB3 yet remains sensitive to the dual EGFR/ErbB2 kinase inhibitor GW572016. Oncogene.

[15] Pedersen K., Angelini P.-D., Laos S., Bach-Faig A., Cunningham M.P., Ferrer-Ramón C., Luque-García A., García-Castillo J., Parra-Palau J.L., Scaltriti M. (2009). A Naturally Occurring HER2 Carboxy-Terminal Fragment Promotes Mammary Tumor Growth and Metastasis. Mol. Cell. Biol..

[16] Lin Y.Z., Clinton G.M. (1991). A soluble protein related to the HER-2 proto-oncogene product is released from human breast carcinoma cells. Oncogene.

[17] Zabrecky J.R., Lam T., McKenzie S.J., Carney W. (1991). The extracellular domain of p185/neu is released from the surface of human breast carcinoma cells, SK-BR-3. J. Biol. Chem..

[18] Kang C.-C., Ward T.M., Bockhorn J., Schiffman C., Huang H., Pegram M.D., Herr A.E. (2018). Electrophoretic cytopathology resolves ERBB2 forms with single-cell resolution. Npj Precis. Oncol..

[19] Arribas J., Parra-Palau J.L., Pedersen K. (2010). HER2 fragmentation and breast cancer stratification. Clin. Cancer Res..

[20] Parra-Palau J.L., Pedersen K., Peg V., Scaltriti M., Angelini P.D., Escorihuela M., Mancilla S., Sanchez Pla A., Ramon Y.C.S., Baselga J. (2010). A major role of p95/611-CTF, a carboxy-terminal fragment of HER2, in the down-modulation of the estrogen receptor in HER2-positive breast cancers. Cancer Res..

[21] Martín-Pérez R., Yerbes R., Mora-Molina R., Cano-González A., Arribas J., Mazzone M., López-Rivas A., Palacios C. (2017). Oncogenic p95HER2/611CTF primes human breast epithelial cells for metabolic stress-induced down-regulation of FLIP and activation of TRAIL-R/Caspase-8-dependent apoptosis. Oncotarget.

[22] Sperinde J., Jin X., Banerjee J., Penuel E., Saha A., Diedrich G., Huang W., Leitzel K., Weidler J., Ali S.M. (2010). Quantitation of p95HER2 in paraffin sections by using a p95-specific antibody and correlation with outcome in a cohort of trastuzumab-treated breast cancer patients. Clin. Cancer Res..

[23] Sáez R., Molina M.A., Ramsey E.E., Rojo F., Keenan E.J., Albanell J., Lluch A., Garcia-Conde J., Baselga J., Clinton G.M. (2006). p95HER-2 predicts worse outcome in patients with HER-2-positive breast cancer. Clin. Cancer Res..

[24] Scaltriti M., Rojo F., Ocana A., Anido J., Guzman M., Cortes J., Di Cosimo S., Matias-Guiu X., Ramon y Cajal S., Arribas J. (2007). Expression of p95HER2, a truncated form of the HER2 receptor, and response to anti-HER2 therapies in breast cancer. J. Natl. Cancer Inst..

[25] Ross J.S., Slodkowska E.A., Symmans W.F., Pusztai L., Ravdin P.M., Hortobagyi G.N. (2009). The HER-2 Receptor and Breast Cancer: Ten Years of Targeted Anti–HER-2 Therapy and Personalized Medicine. Oncologist.

[26] Kocar M., Bozkurtlar E., Telli F., Yumuk F., Kaya H., Kocar H., Turhal N.S. (2014). P95-HER2 and trastuzumab resistance in metastatic breast cancer; is immunohistochemistry appropriate?. J. BUON.

[27] Eliyatkin N.O., Aktas S., Ozgur H., Ercetin P., Kupelioglu A. (2016). The role of p95HER2 in trastuzumab resistance in breast cancer. J. BUON.

[28] Sperinde J., Huang W., Vehtari A., Chenna A., Kellokumpu-Lehtinen P.L., Winslow J., Bono P., Lie Y.S., Petropoulos C.J., Weidler J. (2018). P95HER2 methionine 611 carboxy-terminal fragment is predictive of trastuzumab adjuvant treatment benefit in the fin her trial. Clin. Cancer Res..

[29] Scaltriti M., Chandarlapaty S., Prudkin L., Aura C., Jimenez J., Angelini P.D., Sanchez G., Guzman M., Parra J.L., Ellis C. (2010). Clinical benefit of lapatinib-based therapy in patients with human epidermal growth factor receptor 2-positive breast tumors coexpressing the truncated p95HER2 receptor. Clin. Cancer Res..

[30] Parra-Palau J.L., Morancho B., Peg V., Escorihuela M., Scaltriti M., Vicario R., Zacarias-Fluck M., Pedersen K., Pandiella A., Nuciforo P. (2014). Effect of p95HER2/611CTF on the response to trastuzumab and chemotherapy. J. Natl. Cancer Inst..

[31] Jin Y., Lorvik K.B., Jin Y., Beck C., Sike A., Persiconi I., Kvaloy E., Saatcioglu F., Dunn C., Kyte J.A. (2022). Development of STEAP1 targeting chimeric antigen receptor for adoptive cell therapy against cancer. Mol. Ther. Oncolytics.

[32] Esmaeil Dorraji S., Hovd A.M.K., Kanapathippillai P., Bakland G., Eilertsen G.Ø., Figenschau S.L., Fenton K.A. (2018). Mesenchymal stem cells and T cells in the formation of Tertiary Lymphoid Structures in Lupus Nephritis. Sci. Rep..

[33] Gomes P., Andreu D. (2002). Direct kinetic assay of interactions between small peptides and immobilized antibodies using a surface plasmon resonance biosensor. J. Immunol Methods.

[34] Jumper J., Evans R., Pritzel A., Green T., Figurnov M., Ronneberger O., Tunyasuvunakool K., Bates R., Žídek A., Potapenko A. (2021). Highly accurate protein structure prediction with AlphaFold. Nature.

[35] Leem J., Dunbar J., Georges G., Shi J., Deane C.M. (2016). ABodyBuilder: Automated antibody structure prediction with data–driven accuracy estimation. mAbs.

[36] Pierce B.G., Wiehe K., Hwang H., Kim B.H., Vreven T., Weng Z. (2014). ZDOCK server: Interactive docking prediction of protein-protein complexes and symmetric multimers. Bioinformatics.

[37] Honorato R.V., Koukos P.I., Jiménez-García B., Tsaregorodtsev A., Verlato M., Giachetti A., Rosato A., Bonvin A.M.J.J. (2021). Structural Biology in the Clouds: The WeNMR-EOSC Ecosystem. Front. Mol. Biosci..

[38] Brenke R., Hall D.R., Chuang G.Y., Comeau S.R., Bohnuud T., Beglov D., Schueler-Furman O., Vajda S., Kozakov D. (2012). Application of asymmetric statistical potentials to antibody-protein docking. Bioinformatics.

[39] Mayne L. (2016). Hydrogen Exchange Mass Spectrometry. Methods Enzymol..

[40] Zhang M.M., Huang R.Y.C., Beno B.R., Deyanova E.G., Li J., Chen G., Gross M.L. (2020). Epitope and Paratope Mapping of PD-1/Nivolumab by Mass Spectrometry-Based Hydrogen–Deuterium Exchange, Cross-linking, and Molecular Docking. Anal. Chem..

[41] Grauslund L.R., Calvaresi V., Pansegrau W., Norais N., Rand K.D. (2021). Epitope and Paratope Mapping by HDX-MS Combined with SPR Elucidates the Difference in Bactericidal Activity of Two Anti-NadA Monoclonal Antibodies. J. Am. Soc. Mass Spectrom..

[42] Kabat E.A., Wu T.T. (1971). Attempts to locate complementarity-determining residues in the variable positions of light and heavy chains. Ann. N. Y. Acad. Sci..

[43] Te Wu T., Kabat E.A. (1970). An analysis of the sequences of the variable regions of bence jones proteins and myeloma light chains and their implications for antibody complementarity. J. Exp. Med..

[44] Rawat P., Sharma D., Srivastava A., Janakiraman V., Gromiha M.M. (2021). Exploring antibody repurposing for COVID-19: Beyond presumed roles of therapeutic antibodies. Sci. Rep..

[45] Ruiz I.R., Vicario R., Morancho B., Morales C.B., Arenas E.J., Herter S., Freimoser-Grundschober A., Somandin J., Sam J., Ast O. (2018). P95HER2–T cell bispecific antibody for breast cancer treatment. Sci. Transl. Med..

[46] Ferraro E., Drago J.Z., Modi S. (2021). Implementing antibody-drug conjugates (ADCs) in HER2-positive breast cancer: State of the art and future directions. Breast Cancer Res..

[47] Spector N.L., Blackwell K.L. (2009). Understanding the mechanisms behind trastuzumab therapy for human epidermal growth factor receptor 2-positive breast cancer. J. Clin. Oncol..

[48] Morgan R.A., Yang J.C., Kitano M., Dudley M.E., Laurencot C.M., Rosenberg S.A. (2010). Case report of a serious adverse event following the administration of T cells transduced with a chimeric antigen receptor recognizing ERBB2. Mol. Ther..

[49] Ohnstad H.O., Borgen E., Falk R.S., Lien T.G., Aaserud M., Sveli M.A.T., Kyte J.A., Kristensen V.N., Geitvik G.A., Schlichting E. (2017). Prognostic value of PAM50 and risk of recurrence score in patients with early-stage breast cancer with long-term follow-up. Breast Cancer Res..

