# Supplementary material for: Development of a High-Affinity Antibody against the Tumor-Specific and Hyperactive 611-p95HER2 Isoform
Source: Cancers (Basel). 2022 Oct 5;14(19):4859. doi: 10.3390/cancers14194859 (PMC9563779; doi:10.3390/cancers14194859)
Supplement: Supplementary file 1 [file cancers-14-04859-s001.zip › cancers-1925438-supplementary.pdf]

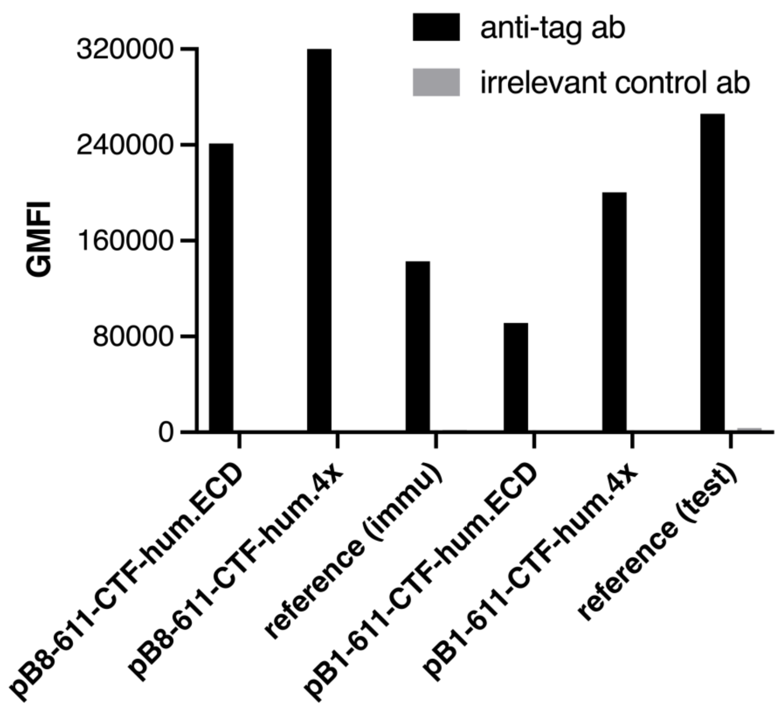

Supplementary Figure S1: Expression of 611-CTF-HER2 in cells used for rat immunization and serum screening.

Development of p95HER2 antibodies was done by immunizing rats with cells expressing 611-CTF-HER2. HEK-293 cells were transfected with different 611-CTF-HER2 constructs. The surface expression of p95HER2 was measured by flow cytometry, using an anti-tag antibody. An irrelevant anti-tag antibody was used as a control. The bar chart shows the global mean flourescence intensity (GMFI) for each construct. The constructs labelled "ECD" contain one repeat of the extracellular domain of 611-CTF-p95HER2, while the constructs labelled "4x" contain four tandem repeats of the extracellular domain to provide more exposure. As shown, the evaluation demonstrated p95HER2 expression of both the pB8 and pB1 constructs on the cell surface. The observed GMFI was higher for the 4x constructs than the ECD constructs, and higher for pB8 (immunization construct) than for pB1 (screening construct).

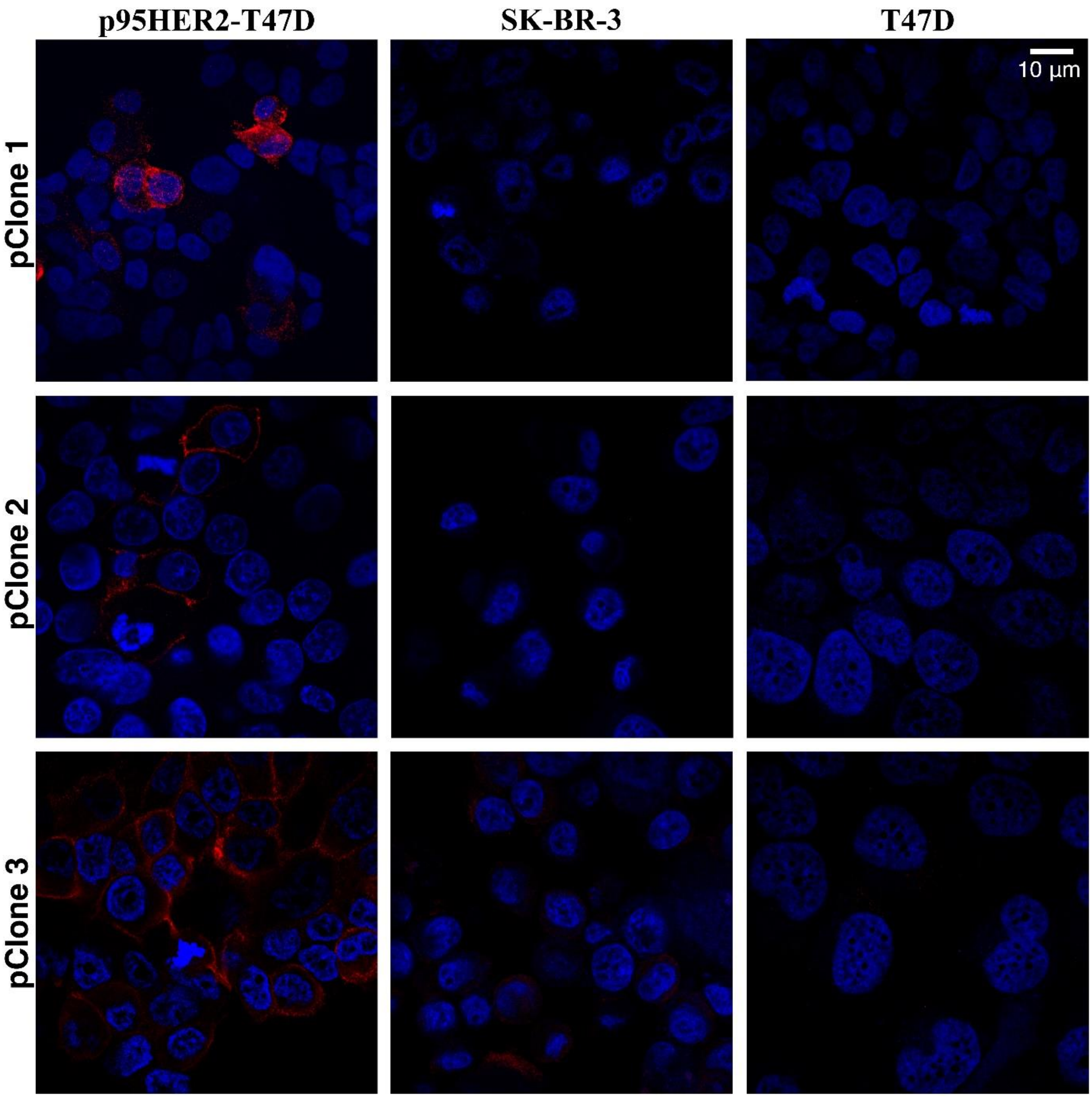

Supplementary Figure S2: Immunofluorescence staining of breast cancer cell lines using polyclonal hybridomas culture supernatants.

The top three polyclonal hybridoma culture supernatants against p95HER2 from early-stage screening, pClones 1, 2, 3, were reactive to p95HER2-T47D, but not to T-47D or SK-BR-3 (full-length HER2+).

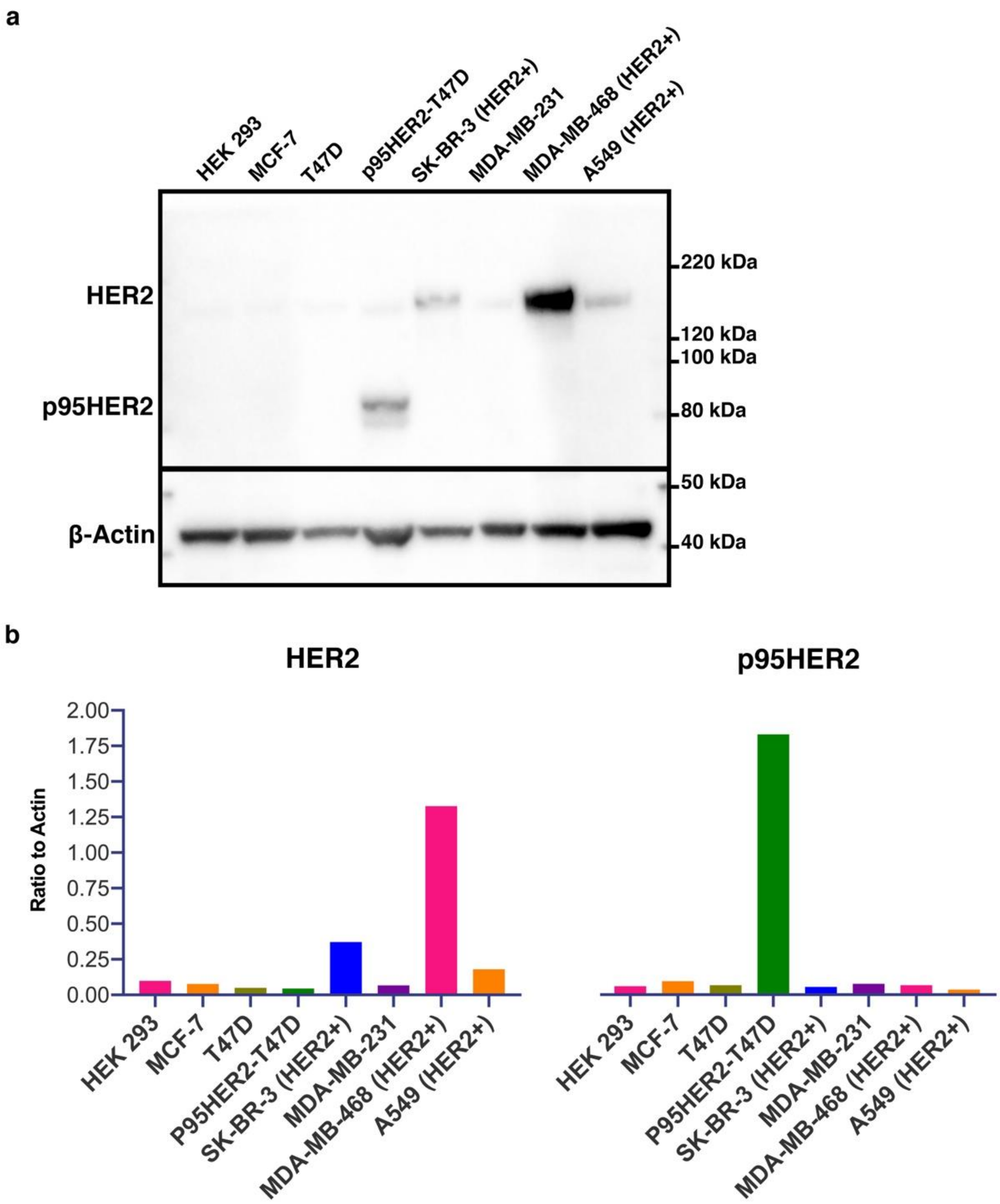

Supplementary Figure S3: p95HER2 and HER2 expression in different cell lines. Western blot was performed on eight cell lines using an antibody against the cytoplasmic domain of HER2. (a) Immunoblotting showing that p95HER2-T47D is the only cell line expressing p95HER2. As expected, SK-BR-3, MDA-MB-468 (transduced with HER2) and A549 express full-length HER2. MDA-MB-468 expresses the highest level of HER2, followed by SK-BR-3. (b) The signal intensity of each band was measured and normalized to actin.

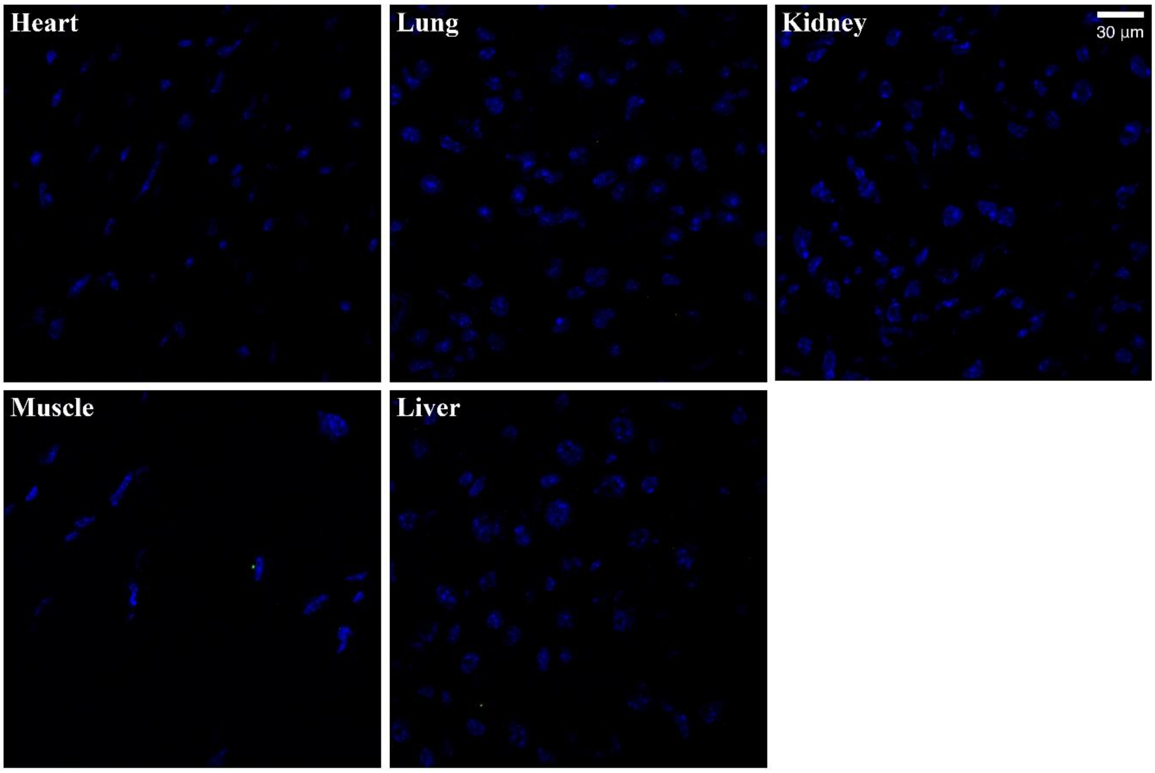

Supplementary Figure S4: Immunofluorescence staining of mouse tissues using Oslo-2 mAb.

Oslo-2 mAb did not show any non-specific binding to normal mouse tissues such as heart, lung, kidney, muscle, and liver and no evidence of cross reactivity was observed.

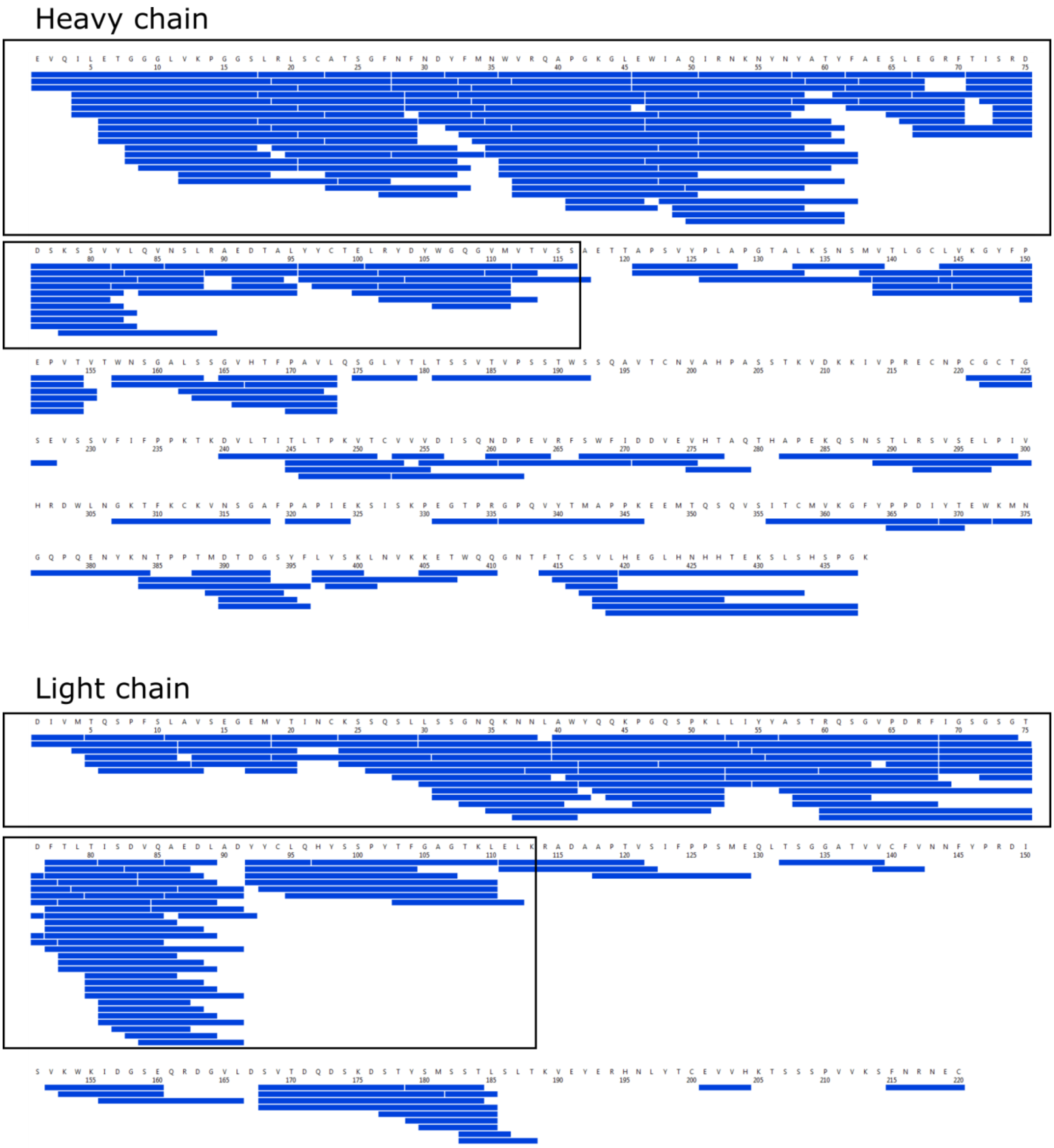

Supplementary Figure S5: Peptide coverage map for HDX experiments.
Peptide pool generated from non-deuterated samples of the Oslo-2 mAb used as a basis for peptide identification in the deuterated samples. Each of the bars represents a single unique peptide. A total of 205 peptides in the heavy chain and 132 peptides in the light chain were identified. The heavy chain and light chain variable domains (in boxes) exhibit 100% sequence coverage and a high degree of overlapping peptides, providing high confidence in HDX results.

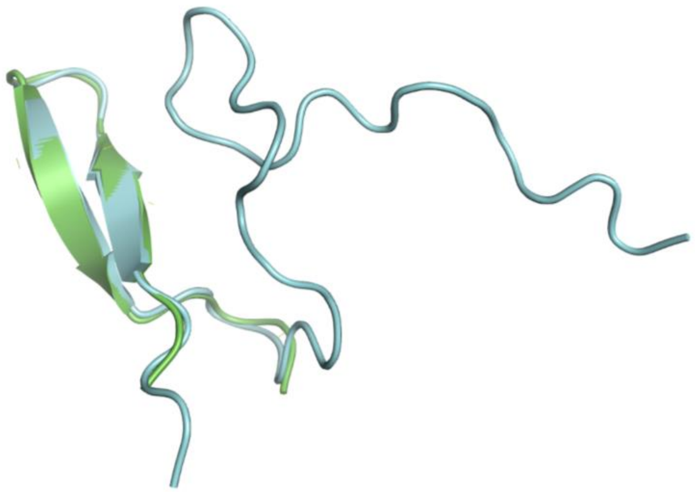

Supplementary Figure S6: Modeling of p95HER2 structure.
Comparison of N-terminal crystal structure of p95HER2 region in HER2 extracellular domain (in green color, Protein Data Bank id. 7MN5) aligned with AlphaFold 2.0 modelled structure of p95HER2 (in blue color, Root Mean Squared Deviation (RMSD): 0.631 Å). α-helices are shown as thick tubes and β-strands as arrows.

	HER2-611-CTF transfected cells	
**Clone**	**GMFI**	**% positive**
1	23615	79 %
2	28840	97 %
3	32606	109 %
4	32320	108 %
5	18319	61 %
6	19448	65 %
7	19550	65 %
8	23022	77 %
9	20111	67 %
10	18106	61 %
11	17718	59 %
12	17579	59 %
13	17311	58 %
14	17196	58 %
15	17079	57 %
16	16947	57 %
17	15589	52 %
18	15260	51 %
19	14739	49 %
20	13655	46 %
21	13422	45 %
22	13217	44 %
23	13030	44 %
24	12927	43 %
**pos Ctr**	29863	100 %
**neg Ctr**		0 %

Supplementary Figure S7: Early-stage polyclonal hybridoma cultures supernatants screening against p95HER2.

Hybridoma supernatants were tested by iQue using mammalian screening +/- 611-CTF-p95HER cell lines. Positive control antibodies were a commercial anti-mouse IgG secondary antibody and 611-CTF-p95HER negative cells were used as negative controls. The top 24 polyclonal hybridoma cultures supernatants candidates are shown. The criterion for clones rated as positive was 4 time over negative control.

	HER2-611-CTF transfected cells		Non-transfected cells
**Clone**	**GMFI**	**% positive**	**GMFI**
1	298630	128 %	1214
2	507000	217 %	107220
3	2356	1 %	2065
**pos Ctr**	233631	100 %	1283
**neg Ctr**	1782	1 %	1073

Supplementary Figure S8: Early-stage monoclonal hybridoma cultures supernatants screening against p95HER2.

The GMFI top 3 candidates selected from polyclonal screening are shown at monoclonal level. The iQue screening revealed only mClone 1 was specific to p95HER2, where mClone 2 showed strong and unspecific binning capacity to p95HER2- cells. mClone 3 was not reactive to p95HER2+ cells, but stained weakly p95HER2- cells unpacifically.

**Paratope**	**Epitope**					
**H38F**	B14Q	B15P				
**H52W**	B3I					
**H50Q**	B3I					
**H57R**	B17P	B4W	B15P	B16C	B3I	B18I
**H61Y**	B14Q	B17P	B16C	B15P		
**H62N**	B19N	B18I	B17P			
**H64A**	B19N					
**H65T**	B1M					
**H66Y**	B4W	B3I	B19N	B2P	B1M	
**H67F**	B1M					
**H69E**	B1M					
**H95L**	B13C	B14Q				
**L31S**	B27L					
**L33G**	B27L	B28D				
**L34N**	B27L	B31G	B7P			
**L36K**	B10E	B9E	B11G	B8D	B7P	
**L38N**	B5K					
**L56Y**	B11G	B12A	B13C			
**L107H**	B5K					
**L108Y**	B24C	B5K				
**L109S**	B5K					
**L114S**	B2P	B1M	B3I			
**L116Y**	B5K	B3I				

Supplementary Table S1: Interaction of each paratope residue with epitope residues within 4.5 Å contact distance.
